# The influence of breast milk microbiota from HIV-infected women on infant gut microbiota colonization within the first two weeks of life

**DOI:** 10.3389/frmbi.2026.1611702

**Published:** 2026-01-28

**Authors:** Privilege Tendai Munjoma, Jacqueline Wyss, Arthur John Mazhandu, Sebastian Bruno Ulrich Jordi, Stephanie Christine Ganal-Vonarburg, Rutendo Zinyama-Gutsire, Leolin Katsidzira, Bahtiyar Yilmaz, Benjamin Misselwitz, Kerina Duri

**Affiliations:** 1 Immunology Unit, Department of Laboratory Diagnostic and Investigative Sciences, University of Zimbabwe Faculty of Medicine and Health Sciences (UZ-FMHS), Harare, Zimbabwe; 2 Department of Visceral Surgery and Medicine, Inselspital, Bern University Hospital, University of Bern, Bern, Switzerland; 3 Department for Biomedical Research, Maurice Müller Laboratories, University of Bern, Bern, Switzerland; 4 Department of Internal Medicine, University of Zimbabwe Faculty of Medicine and Health Sciences (UZ-FMHS), Harare, Zimbabwe; 5 Medizinische Klinik 2, University Hospital Ludwig Maximilian University of Munich, Munich, Germany

**Keywords:** breast milk, breast milk microbiota, HIV infection, infant gut microbiota, maternal gut microbiota

## Abstract

**Background:**

The human milk microbiota significantly contributes to the shaping of the infant gut microbiota during early life. Influenced by maternal factors such as birth mode, diet, and breastfeeding practices, these microbial communities are critical for infant health. We explored the effect of maternal human immunodeficiency virus (HIV) status and breastfeeding practices on breast milk microbiota composition and its correlation with infant gut microbiota between 7 and 14 days postpartum.

**Methods:**

Breast milk and stool microbiota from 68 lactating HIV-infected and uninfected women and their 69 infants (including one set of twins) were characterized using 16S rRNA gene sequencing. Sociodemographic and clinical data were also collected.

**Results:**

Breast milk microbiota was dominated by *Streptococcus and Gemella*, whereas the infant gut microbiota showed a co-occurrence of early colonizers such as *Enterobacteriaceae_unclassified, Bifidobacterium*, and *Streptococcus*. In contrast, maternal stool exhibited greater microbial diversity, enriched in *Romboutsia* and *Clostridium_sensu_stricto_1*. Small, non-significant differences were observed in alpha diversity by maternal HIV status (Cohen’s d ≈ −0.38; 95% CI: −3.88 to 0.07), suggesting possible modest to no effects, even though *p-values* were not significant. *Clostridium_sensu_stricto_1* was more abundant in HIV-uninfected mothers. Infant HIV exposure and maternal antibiotic prophylaxis had no detectable effect on gut microbiota diversity or composition. Notably, positive correlations were observed between breast milk and infant gut taxa abundances, including *Gemella* (*ρ* = 0.33, *p* = 0.010) and *Enterobacteriaceae_unclassified* (*ρ* = 0.31, *p* = 0.016). SourceTracker analysis indicated that 31.5% of infant gut taxa were traceable to breast milk, with higher contributions in HIV-exposed infants (41%) compared with HIV-unexposed infants (25.6%).

**Conclusion:**

This study is among the first to investigate breast milk microbiota in the context of HIV infection in Zimbabwe. We demonstrated that maternal HIV infection and cotrimoxazole prophylaxis did not measurably alter breast milk or early infant gut microbiota composition. Despite limited statistical power to detect small-to-moderate effects, taxa-level correlations and microbial source tracking supported breast milk as a major contributor to early gut colonization. These results underscore breast milk–mediated microbial seeding in early life, while highlighting the need for larger longitudinal studies to define how maternal HIV status may subtly influence vertical microbial transfer.

## Introduction

Gut microbial colonization initiates at birth and unfolds through a co-evolved host–microbe relationship that supports early-life health. The early-life period is crucial for the initial programming of the infant gut, with potential implications for both short- and long-term health (Boudry et al., 2021). The establishment of the infant gut microbiota is shaped by delivery mode, environmental exposures, maternal diet, lactation, and antibiotic use (Padilha et al., 2019; Boudry et al., 2021; Cortes-Macías et al., 2021; Chong et al., 2022; Liu et al., 2022). Vertical transmission from maternal microbial communities, including vaginal, stool, skin, and breast milk sources, supports this early colonization (Milani et al., 2017), with host and microbial factors jointly influencing community assembly (Gregory et al., 2016; Drell et al., 2017; Hermansson et al., 2019). Breastfeeding, in particular, promotes a characteristic early-life microbiota enriched in *Bifidobacterium* and supported by human milk oligosaccharides (HMOs) (Jost et al., 2015; Milani et al., 2017; Sánchez et al., 2021; Cheema et al., 2022; Chong et al., 2022; Dinleyici et al., 2023; Froń and Orczyk-Pawiłowicz, 2024; Sun et al., 2025). Breastfeeding practices also vary by maternal HIV status, with higher rates of exclusive breastfeeding among HIV-infected women (Diakhate et al., 2024).

The role of mother–infant interaction in supporting the development of the infant microbiota remains an area of ongoing investigation. Several studies have indicated a co-occurrence of bacteria in breast milk and the infant gut over the lactation period, with shared microbial taxa such as *Streptococcus* and *Bifidobacterium* (Fehr et al., 2020; Laursen et al., 2021; Ding et al., 2022; Haddad et al., 2022; Martínez-Martínez et al., 2024). This suggests that breast milk–associated bacteria transmitted during breastfeeding may influence infant gut development. There is a lack of studies on breast milk microbiota and infant gut colonization in Zimbabwe and sub-Saharan Africa. Complex microbial interactions occur between mothers and infants, with the breast milk microbiota closely resembling the infant oral microbiota but differing from the infant gut microbiota (Williams et al., 2019). During early life, the infant gut microbiota exhibits lower diversity compared with the maternal gut microbiota. A Croatian study demonstrated higher phylogenetic diversity and compositional differences in the infant gut relative to breast milk (Banić et al., 2022). In a multi-site study across Europe, North America, and Africa, breast milk and infant gut microbiota differed in community membership and structure, although significant correlations were found between some breast milk and infant stool taxa (Pace et al., 2021).

Studies from different populations have identified correlations between breast milk and infant gut taxa, but findings vary across settings (Fehr et al., 2020; Ding et al., 2022; Li et al., 2022; Wang et al., 2023). Bacteria such as *Lactobacillus*, *Streptococcus, Bifidobacterium, Staphylococcus*, and *Enterococcus* have been found to co-occur between breast milk, infant oral, and gut environments (Pace et al., 2021). In a Chinese study, breast milk *Lactobacillus* correlated with *Bifidobacterium* and *Clostridium* in infant stool during the first month of life (Li et al., 2022). This highlights the need for disease-, region-, and ethnicity-specific microbiome investigations to better understand factors shaping the infant gut microbiota and their implications for health (Pheeha et al., 2023). To our knowledge, no studies have investigated breast milk microbiota and its contribution to infant gut colonization in Zimbabwe, where HIV prevalence is high (Zhou et al., 2023). HIV infection is associated with systemic inflammation and altered immune responses in breastfeeding women, which can affect the microbial environment of breast milk. Antiretroviral therapy (ART) may also indirectly shape the breast milk microbiome through modification of maternal physiology or through exposure in breast milk. Breast milk is a major source of microbes for the infant gut, and perturbations in maternal milk microbiota due to HIV, ART, or antibiotic use could influence infant gut colonization (Maqsood et al., 2022; Tobin et al., 2024). Altered breast milk microbial diversity in lactating women living with HIV is a potential driver of the immune dysregulation observed in HIV-exposed infants.

In this study, we characterized the breast milk and infant gut microbiota of HIV-infected and uninfected mother–infant pairs between 7 and 14 days postpartum using 16S rRNA gene sequencing. By comparing microbial diversity and community composition across these groups, and linking paired breast milk–infant stool profiles, we investigated whether maternal HIV status or prophylactic antibiotic use alters early microbial assembly or the transfer of milk-associated taxa to the infant gut.

## Materials and methods

### Study design

This cross-sectional study was nested within the University of Zimbabwe Birth Cohort Study (UZBCS), which has been previously described and is registered at www.clinicaltrials.gov (NCT04087239) (Duri et al., 2020). Women and their infants received postnatal care services at four primary healthcare clinics located in high-density areas of Harare, Zimbabwe, namely Budiriro, Glenview, Kuwadzana, and Rujeko clinics.

### Inclusion and exclusion

Participants were enrolled during antenatal care visits and were required to be 18 years or older and at least 20 weeks’ gestation. All women provided written informed consent for their participation and parental consent for their infants. Women were required to be willing to give birth at one of the study sites and to be followed up as a mother–infant pair. Women who consented to be tested or retested for HIV and assessed for health disorders were included. Women with severe mental health conditions or other comorbidities that could impair participation were excluded. Infants with health disorders (birth complications or congenital disorders) that prevented adherence to study procedures were excluded from the study. Since stool samples were not collected before 2019, only women enrolled in the UZBCS from 2019 onward were included in this analysis. Both women and their infants were evaluated between 7 and 14 days postpartum. A total of 26 HIV-infected and 42 HIV-uninfected lactating women, along with their 69 infants (including one set of twins), were studied.

### Data collection

Participant data were collected using IRB-approved questionnaires and entered into a Research Electronic Data Capture (REDCap) database, a secure platform for research data management (Harris et al., 2019). Physical examinations, including anthropometric assessments, were carried out for mother–infant pairs by trained, qualified nurses.

### Infant HIV screening and morbidity documentation

All HIV-infected women in this study were taking combination antiretroviral therapy (cART) and used a formulation of efavirenz, lamivudine, and tenofovir disoproxil fumarate (Tenolam-E), following World Health Organization (WHO) guidelines (Update of recommendations on first- and second-line antiretroviral regimens, 2023). Dried blood spots were collected on Whatman filter paper from EDTA whole blood and used for screening HIV-exposed infants (infants born to HIV-infected mothers) for HIV infection. The methods used for early infant diagnosis (EID) of HIV have been previously described (Duri et al., 2020). Infants who either fell ill, were hospitalized, or received any antibiotics prior to the collection of biosamples during the study visit were documented. Illnesses were grouped into fever/flu, diarrhea, skin conditions, acute respiratory infections, and eye conditions. Combinations of these illnesses were also documented.

### Fecal sample collection, DNA extraction, and 16S rRNA sequencing

Maternal stool samples were collected at a single time point between 7 and 14 days postpartum using sterile 50 mL sample containers and subsequently aliquoted into 2 mL tubes for storage at −80°C until laboratory analysis. Infant stool samples were concurrently obtained from diapers on the same day using sterile tongue depressors to transfer samples into sterile 25 mL sample containers. Aliquots of 2 mL were prepared and stored at −80°C until laboratory analysis. Approximately 250 mg of stool was used for DNA extraction using a QIAamp PowerFecal Pro DNA Kit (Qiagen, Dusseldorf, Germany) as previously described (Yilmaz et al., 2022; Munjoma et al., 2023). In brief, fecal bacterial DNA was eluted in 70 μL of elution buffer and stored at −20°C prior to amplification. DNA amplification targeting the V5–V6 regions of the 16S rRNA gene was performed using bacteria-specific primers (forward primer: 5′-CCATCTCATCCCTGCGTGTCTCCGACTCAGC-barcode-ATTAGATACCCYGGTAGTCC 3’; reverse primer: 5’ CCTCTCTATGGGCAGTCGGTGATA CGAGCTGACGACARCCATG-3’) as previously described (Yilmaz et al., 2018). The polymerase chain reaction (PCR) protocol consisted of an initial denaturation at 94°C for 5 min, followed by 35 cycles of denaturation at 94°C for 1 min, annealing at 46°C for 20 s, elongation at 72°C for 30 s, and a final elongation step at 72°C for 7 min (Yilmaz et al., 2019).

PCR products were run on a 1% agarose gel, and an amplicon of approximately 350 bp was expected. Amplicons were purified using the QIAquick Gel Extraction Kit (Qiagen, Dusseldorf, Germany), and concentrations were determined using a Qubit double-stranded DNA (dsDNA) high-sensitivity (HS) Assay Kit (Invitrogen) on a Qubit 3.0 Fluorometer (Thermo Fisher Scientific). Amplicon concentrations were normalized to 26 pM for sequencing library preparation and sequenced on an Ion Torrent PGM™ System (Thermo Fisher Scientific) using the Ion PGM™ Sequencing Kit as previously described (Whiteley et al., 2012).

### Breast milk sample collection, DNA extraction, and 16S rRNA sequencing

Maternal whole breast milk samples were collected on the same day as maternal and infant stool samples and stored on ice prior to shipment to the laboratory, where sample processing was performed within 6 h of collection. In brief, whole breast milk was collected through manual expression by lactating women into sterile 15 mL containers. Milk samples were dispensed into 1.8 mL tubes and subsequently stored at −80°C in preparation for laboratory analysis. Prior to breast milk expression, no sanitization procedures were implemented, ensuring a more accurate representation of bacterial exposure from breast milk to infants. Breast milk bacterial DNA extraction was carried out from 1.0 mL of whole breast milk using the QIAamp PowerFecal Pro DNA Kit (Qiagen, Dusseldorf, Germany) with modifications to the initial steps of the protocol. The modifications included the following steps.

Maternal whole milk was thawed on ice and gently mixed by aspirating and dispensing using a pipette. An aliquot of 1.0 mL of whole milk was taken from the parent tube and centrifuged at 10,000 × g for 10 min at 4°C. The fat layer was removed using sterile loops, and the supernatant was decanted. The pellet was resuspended using lysis buffer (provided with the kit), and glass beads were added for bead beating as per the manufacturer’s specifications. The remaining steps following bead beating were carried out according to the manufacturer’s protocol, except for the DNA elution step, which was performed using 30 μL of elution buffer. Extracted DNA was stored at −20°C prior to amplification. DNA extracted from milk samples was prepared for sequencing as described in the previous section. Given that breast milk contains low amounts of microbial DNA, potential contamination was controlled by using PCR negative controls, which were assessed for amplification of the target region on a 1% agarose gel. However, PCR negative controls were not sequenced, and no contamination filtering for breast milk samples was conducted for downstream analysis.

### Data analysis

#### Participant sociodemographic and clinical characteristics

Data were analyzed using R software, version 4.2.2 (R: the R project for statistical computing, 2024). Continuous variables were tested for normality using the Shapiro–Wilk test and summarized as median with interquartile range (IQR) or mean ± standard deviation (SD), as appropriate. Group comparisons of continuous variables were performed using the Mann–Whitney U test, Kruskal–Wallis test, Wilcoxon signed-rank test, or Student’s t-test, as appropriate. Counts and percentages were used for reporting categorical data, and associations were determined using either Fisher’s exact test or the chi-squared test.

#### Analysis of stool and breast milk 16S rRNA microbial data

As previously described, FASTQ sequencing files from the Ion Torrent PGM™ System were processed using the Quantitative Insights into Microbial Ecology 2 (QIIME 2), version 2021.11, pipeline (Caporaso et al., 2010; Yilmaz et al., 2018; Bolyen et al., 2019). Amplicon sequence variants were assigned taxonomy at the 97% sequence identity threshold using the QIIME 2 *q2-feature-classifier* plugin and a naïve Bayes classifier. Taxonomic assignment was performed using the SILVA reference database (Quast et al., 2013).

A feature table and mapping file were used to generate a phyloseq object using the R package *phyloseq* (McMurdie and Holmes, 2013; Callahan et al., 2016). Samples with ≥2,000 sequence reads were included for further analysis. A rarefaction curve was generated in R to assess whether sequencing depth reached saturation. Sequencing reads were rarefied to an even depth corresponding to the lowest number of sequencing reads (2,088 reads). Further diversity and downstream analyses were conducted using non-rarefied sequence data. Diversity within communities was determined using alpha diversity indices (Simpson and Shannon indices), and inter-community diversity was determined using beta diversity measures (Bray–Curtis dissimilarity and unweighted UniFrac) (Callahan et al., 2016). Cohen’s d was calculated with Hedges’ correction for small samples to determine the effect of HIV infection on microbial diversity. Mann–Whitney U and Kruskal–Wallis tests were used to assess the significance of differences in alpha diversity between groups. Permutational multivariate analysis of variance (PERMANOVA) and pairwise Adonis tests were used to assess the significance of differences in beta diversity between groups (Arbizu, 2024). The permutation test for homogeneity of dispersion (PERMDISP) was used to minimize confounding by within-group variability. For pairwise comparisons of dispersion between groups, Tukey’s HSD was used. Linear discriminant analysis effect size (LEfSe) was used to identify significant differences in bacterial taxon relative abundances across sample types. A linear discriminant analysis (LDA) score cutoff of 3.5 was used to estimate effect size, and taxa significant at *p* < 0.05 were reported.

Multivariable Association with Linear Models (MaAsLin2) and multivariable linear regression were used to assess associations between gut microbiota abundance and categorical or continuous variables (e.g., age, antibiotic use, and HIV status) (Mallick et al., 2021). *q*-values were calculated for multiple testing correction using the Benjamini–Hochberg (BH) false discovery rate (FDR). Taxa significant at *q* < 0.05 were reported. Figures were generated in R, with color and font subsequently edited in Adobe Illustrator, version 27.3.1. Spearman’s *rho* correlation (*ρ*) was used to assess correlations of relative abundances between sample types and further examined using canonical correlation analysis (CCA). CCA assesses associations between multivariate sets of variables by identifying linear combinations that maximize correlations between groups. The *cancor()* function from the CCA package in R was used after loading a taxonomy file containing taxa relative abundance data. Data were standardized using the scale() function to equalize variance across features. All paired breast milk and infant stool data were included in the analysis. The analysis was conducted for core taxa, where the taxonomy file was subset by sample type and merged to match breast milk and infant stool taxa abundances for mother–infant pairs. Relative abundances were correlated, and the corresponding coefficients for breast milk and infant stool were used to calculate correlations for canonical covariate pairs, which were verified by comparison with *cancor*() output. In addition, the effects of maternal HIV status, maternal age, infant age group, and antibiotic use on the covariates were assessed.

Shared core taxa between breast milk and infant stool were identified, with core taxa defined as those with a relative abundance of ≥0.1% and present in at least 50% of all samples. These thresholds were selected to (i) minimize inclusion of low-count artifacts and (ii) balance stability with inclusivity. Comparative evaluations of core microbiome methods indicate that no universal standard exists, and thresholds should be justified based on study goals and data characteristics (Sharon et al., 2022; Custer et al., 2023). Furthermore, *SourceTracker* analysis was performed using QIIME 2 (version 2020.6) and *SourceTracker* (version 2.0.1) to estimate the proportion of infant stool taxa originating from breast milk (Knights et al., 2011). Estimates were also generated for the proportion of core taxa attributed to breast milk versus other uncharacterized sources. *SourceTracker* provides probabilistic estimates rather than direct evidence of microbial transfer and should therefore be interpreted cautiously. A feature table with counts, a metadata file, and a taxonomy file were imported into QIIME 2. In the metadata file, source and sink variables were clearly identified, with breast milk designated as the source and infant stool as the sink. Per-sink feature assignments were determined for each pair. Interactive bar plot outputs were viewed in QIIME 2 View to examine feature contributions.

## Results

### Participant sociodemographic and clinical data

A total of 68 lactating women and their 69 infants (including one set of twins), seen once as a mother–infant pair between 7 and 14 days postpartum, were included in this study. Maternal sociodemographic characteristics and clinical data are shown in **Table 1**. HIV-infected lactating women were older (median age, 32 years; IQR, 29-36) compared with HIV-uninfected women (median age, 26 years; IQR, 21–29) (*p* < 0.001). Among all lactating women, 7.4% reported cracked nipples, and 98.5% were exclusively breastfeeding their infants. The HIV-infected group demonstrated higher levels of food insecurity, with 42.3% reporting eating fewer meals per day due to insufficient food, compared with 11.9% of HIV-uninfected women (*p* = 0.007).

**Table 1:** Maternal sociodemographic and clinical characteristics during pregnancy and at 7–14 days postpartum (*n* = 68), stratified by HIV status.

Variable	HIV-infected (*n* = 26)	HIV-uninfected (*n* = 42)	p-value
Socio-demographics during pregnancy			
**Age (years)** [median (IQR)]	32 (29–36)	26 (21–29)	**0.00016**
Marital Status			
Married	24 (92.3%)	42 (100%)	0.083
Widowed/Single	2 (7.7%)	0	
Education			
Completed at least Secondary school	16 (61.5%)	38 (90.5%)	**0.014**
Below Secondary school	10 (38.5%)	4 (9.5%)	
Employment status			
Employed	10 (38.5%)	12 (28.6%)	0.509
Unemployed	16 (61.5%)	30 (71.4%)	
Average family monthly income (USD$)			
[median (IQR)]	400 (268–575)	325 (250-437)	0.299
**Household size** [median (IQR)]	4.0 (3.3-5.0)	4.0 (3.0-5.0)	0.294
**Number of rooms used in house** [median (IQR)]	2 (1-2)	1 (1-2)	0.134
Toilet facility			
Outside house	26 (100%)	42 (100%)	**-**
Inside house	0	0	
**Households sharing toilet facility** [median (IQR)]	2 (1-4)	3 (2-4)	0.184
Sewer bursts frequency			
Never	17 (65.4%)	22 (52.4%)	0.627
Once per year	4 (15.4%)	10 (23.8%)	
Once per month	5 (19.2%)	8 (19.0%)	
Always	0	2 (4.8%)	
Postpartum breastfeeding and complications			
Breast feeding complications			
None	24 (92.3%)	39 (92.9%)	1
Cracked nipples	2 (7.7)	3 (7.1%)	
Breastfeeding status			
Exclusive	26 (100%)	41 (97.6%)	1
Mixed	0	1 (2.4%)	
Postpartum food security			
Worried due to not enough food			
Yes	9 (34.6%)	6 (14.3%)	0.071
No	17 (65.4%)	36 (85.7%)	
Eat non-preferred foods due to lack of resources			
Yes	12 (46.2%)	7 (16.7%)	**0.012**
No	14 (53.8%)	35 (83.3%)	
Eat fewer meals per day due to not enough food			
Yes	11 (42.3%)	5 (11.9%)	**0.007**
No	15 (57.7%)	37 (88.1%)	
Postpartum concurrent medication			
Anti-acids			
Yes	0	1 (2.4%)	1
No	26 (100%)	41 (97.6%)	
Antibiotics			
Yes	12 (46.2%)	2 (4.8%)	**7.10E-05**
No	14 (53.8%)	40 (95.2%)	
Combination ART			
Yes	26 (100%)	NA	–
No	0		
Postpartum clinical data			
**Weight (kg)** [median (IQR)]	62.6 (55.7-69.5)	58.5 (53.5-64.3)	0.113
**MUAC (cm)** [median (IQR)]	25.5 (25.0-26.8)	24.9 (23.5-26.8)	0.135
**Systolic BP (mmHg)** [median (IQR)]	120 (110-120)	110 (110-120)	0.145
**Diastolic BP (mmHg)** [median (IQR)]	80 (70-90)	70 (70-80)	**0.048**
Maternal hospitalisation after delivery			
Yes	0	1 (2.4%)	1
No	26 (100%)	(infant jaundice)	
		41 (97.6%)	

Data are presented as median (IQR) for continuous variables and *n* (%) for categorical variables.

IQR: interquartile range; ART, antiretroviral therapy; BP, blood pressure; MUAC, mid-upper arm circumference; HIV, human immunodeficiency virus. Statistical analysis: Group comparisons were performed using the Mann–Whitney U test or Fisher’s exact test, as appropriate. P-values shown in bold are statistically significant at p<0.05.

All HIV-infected women were receiving cART comprising a triple regimen of tenofovir, lamivudine, and efavirenz (TENOLAM-E) and had no symptoms of acquired immunodeficiency syndrome (AIDS). However, 46.2% of HIV-infected lactating women were taking prophylactic cotrimoxazole (*p* < 0.001), as shown in **Table 1**. Notably, a higher proportion of HIV-uninfected women (90.5%) had completed secondary education (*p* = 0.014), and the majority of both HIV-infected (61.5%) and HIV-uninfected (71.4%) women were unemployed. There were no significant differences in average family income, household size, number of rooms used, or households sharing a toilet in the study population.

The infant sociodemographic and clinical characteristics are shown in **Table 2**. None of the HIV-exposed infants (born to HIV-infected women) were infected with HIV by 7–14 days postpartum. All infants were exclusively breastfed except for one HIV-unexposed uninfected (HUU) infant who was both breastfed and received water. There were no significant differences in infant weight, length, or head circumference by HIV exposure status. A possible association with borderline significance (*p* = 0.055) was observed between mode of delivery and infant HIV exposure, whereby 11.1% of cesarean sections were reported among HIV-exposed uninfected (HEU) infants compared with 0% among their HUU peers.

**Table 2:** Infant sociodemographic and clinical characteristics at 7–14 days postpartum (n = 69, including one set of twins), stratified by HIV exposure status.

Variable	HIV-exposed uninfected (HEU, *n* = 27), including 1 set of twins	HIV-unexposed uninfected (HUU, *n* = 42)	p-value
Sociodemographic data			
**Age (days)** [median (IQR)]	8 (7-11)	7 (7-10)	0.6
Age group			
≤7 days	15 (55.6%)	23 (54.8%)	0.465
>7 days	12 (44.4%)	19 (45.2%)	
Infant sex			
Male	16 (59.3%)	19 (45.2%)	0.326
Female	11 (40.7%)	23 (54.8%)	
Infant baths with soap			
Yes	19 (70.4%)	28 (66.7%)	0.797
No	8 (29.6%)	14 (33.3%)	
Infant currently living with a smoker			
Yes	6 (22.2%)	9 (21.4%)	1
No	21 (77.8%)	33 (78.6%)	
Feeding practices			
Infant breastfeeding			
Yes	27 (100%)	42 (100%)	–
No	0	0	
Infant given formula milk			
Yes	0	0	–
No	27 (100%)	42 (100%)	
Infant given other fluids besides breast milk			
Yes	0	1 (2.4%) (water)	1
No	27 (100%)	41 (97.6%)	
Infant given liquids and semisolids other than breast or formula milk			
Yes	0	0	–
No	27 (100%)	42 (100%)	
Infant given porridge			
Yes	0	0	–
No	27 (100%)	42 (100%)	
Infant given adult diet/lumps and solids			
Yes	0	0	–
No	27 (100%)	42 (100%)	
Clinical data			
Mode of delivery			
Spontaneous normal	24 (88.9%)	42 (100%)	0.055
Caesarean section	3 (11.1%)	0	
**Birth Apgar score (5 min) [**median (IQR)]	9.0 (8.0-9.0)	9.0 (8.25-9.0)	0.549
Birth weight			
<2500g	1 (3.7%)	1 (2.4%)	1
≥2500g	26 (96.3%)	41 (97.6%)	
**Current Weight (kg) [**median (IQR)]	3.1 (2.9-3.4)	3.2 (2.8-3.4)	0.964
**Current Length (cm) [**median (IQR)]	50 (48.6-51.0)	49.0 (48.8-50.3)	0.38
Current Head circumference (cm)			
[median (IQR)]	36.0 (35.5-36.0)	36.0 (35.8-36.0)	0.927
Received BCG vaccine			
Yes	24 (88.9%)	38 (90.5%)	1
No	3 (11.1%)	4 (9.5%)	
Hospital admission since birth			
Yes	0	1 (2.4%) (jaundice)	1
No	27 (100%)	41 (97.6%)	

Data are presented as median (IQR) for continuous variables and n (%) for categorical variables.

BCG, Bacille Calmette–Guérin; IQR, interquartile range; HIV, human immunodeficiency virus. Statistical analysis: Group comparisons were performed using the Mann–Whitney U test or Fisher’s exact test, as appropriate. P-values shown in bold are statistically significant at p<0.05.

### Comparison between the mother stool, breast milk, and infant gut microbiota

Gut microbiota assessment was conducted using stool samples from 65/68 (95.6%) lactating women and 66/69 (95.7%) infants. Three women and three infants from different mother–infant pairs could not provide stool samples on the day of the study visit. Breast milk microbiota was assessed from 62/66 (93.9%) breast milk samples; the remaining four samples were excluded due to low-quality reads (<2,000 reads). To demonstrate that taxonomic assignment reached saturation, a rarefaction curve was generated after rarefying to an even depth corresponding to the lowest number of reads (2,088 reads) (**Supplementary Figure 1**). However, to avoid excluding rare and low-abundance taxa, downstream analyses were conducted on non-rarefied sequence data.

To compare overall richness, evenness, and composition of microbial taxa across sample types (breast milk, maternal stool, and infant stool), alpha diversity measures (Shannon and Simpson indices) were calculated. Significant differences in Shannon and Simpson indices (*p* < 0.05) were observed when comparing pairs of the three sample types (**Supplementary Figure 2A**). Maternal stool and breast milk exhibited higher microbial richness compared with infant stool. We further computed multivariate and linear regression models to determine whether maternal age, weight, antibiotic use, eating fewer meals per day, or Shannon and Simpson indices were associated with HIV infection; none of these variables were significant before or after parameter elimination. Overall microbial composition across the three sample types was compared using Bray–Curtis dissimilarity and UniFrac distance. Significant differences (*p* < 0.001) were observed for both metrics, with distinct clustering and minimal overlap on principal coordinates analysis (PCoA) plots between the three sample types (**Supplementary Figure 2B**). Multiple comparisons using pairwise Adonis tests with Bonferroni correction were performed to assess differences in beta diversity among pairs of sample types.

Significant differences in beta diversity were observed for all paired comparisons after multiple testing adjustment (all *p* = 0.003). Pairwise differences in dispersion (variance) between sample types were assessed using PERMDISP. Maternal milk vs infant stool and maternal stool vs infant stool differed in dispersion (*p* = 0.002 and *p* < 0.001, respectively), whereas maternal stool and maternal milk did not differ (*p* = 0.409).

### Comparison between the mother stool, breast milk, and infant gut microbiota by HIV infection and exposure status

**Figure 1:**
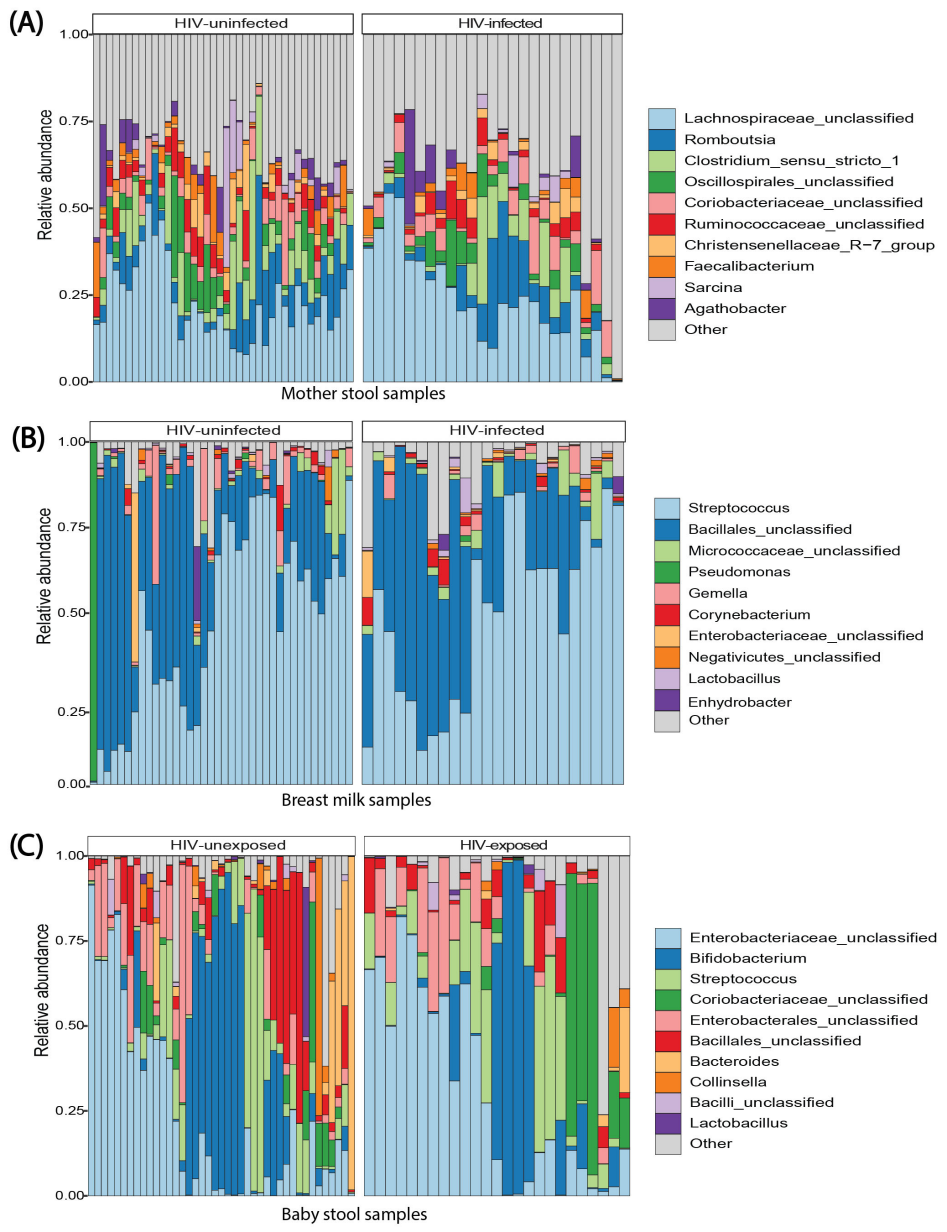
Taxonomy profile of mother stool, breast milk, and infant stool. Bar plots comparing relative abundances of the top 10 most abundant bacterial genera in **(A)** mother stool, **(B)** breast milk, and **(C)** infant stool by HIV infection and exposure status. Each genus is quantified and represented as a percentage of the total microbial content in each sample type.

At the phylum level, Bacillota (83.5%, 89.3%, 27.2%), Actinomycetota (11.3%, 5.3%, 29.7%), Bacteroidota (2.7%, 0.72%, 5.5%), and Pseudomonadota (2.2%, 5.5%, 37.7%) were the most abundant taxa in maternal stool, breast milk, and infant stool, respectively. The top 10 most abundant genera in maternal stool, breast milk, and infant stool were stratified by maternal HIV infection (maternal samples) and infant HIV exposure (infant stool), as shown in **Figure 1**. Overall, *Lachnospiraceae_unclassified*, *Romboutsia*, and *Clostridium_sensu_stricto_1* were among the top 10 most abundant genera in maternal stool, though less prevalent in breast milk and infant stool (**Figure 1A**). *Streptococcus* and Bacillales_*unclassified* were among the most abundant genera in breast milk, though less prevalent in the other sample types (**Figure 1B**). In infant stool, *Streptococcus*, Bacillales_*unclassified*, *Bifidobacterium*, and *Enterobacteriaceae_unclassified* were among the top 10 most abundant genera (**Figure 1C**).

When considering only identified genera, *Romboutsia* (14.9%), *Clostridium_sensu_stricto_1* (12.7%), *Faecalibacterium* (5.7%), and *Agathobacter* (5.5%) were among the most abundant in maternal stool. In breast milk, the most abundant identified genera included *Streptococcus* (80.6%), *Gemella* (5.3%), *Corynebacterium* (2.8%), *Pseudomonas* (2.0%), and *Lactobacillus* (1.5%). The most abundant identified genera in infant stool included *Streptococcus* (38.3%), *Bifidobacterium* (35.6%), *Bacteroides* (7.9%), *Collinsella* (5.6%), and *Lactobacillus* (2.2%) (**Supplementary Figure 3**).

### Mother (stool and breast milk) microbiota and infant gut microbiota comparison by HIV infection and exposure status

**Figure 2:**
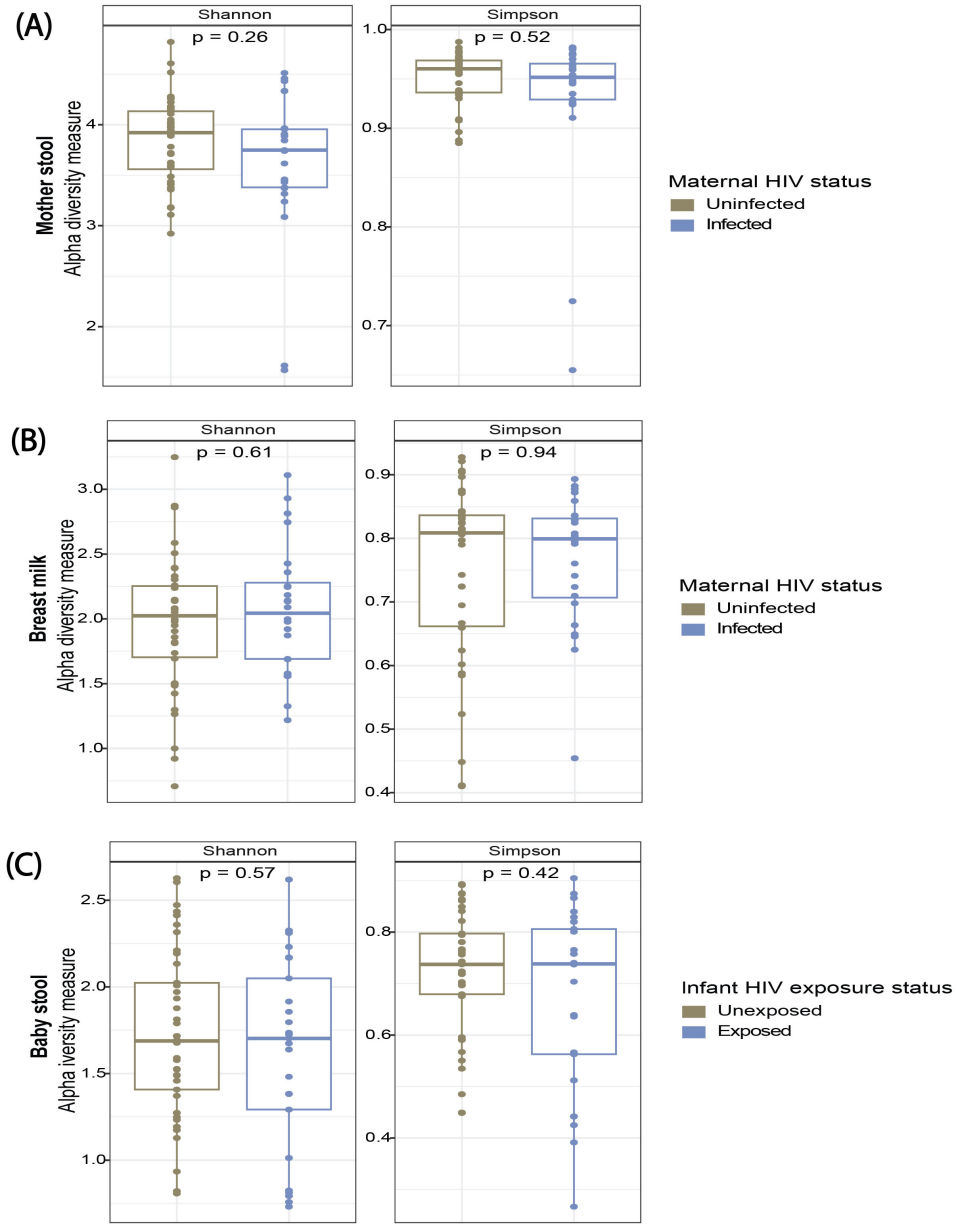
Similar alpha diversity by HIV infection and exposure status. Comparison of alpha diversity indices (Shannon and Simpson indices) in **(A)** mother stool, **(B)** breast milk, and **(C)** infant stool by HIV infection and maternal HIV exposure status.

Alpha and beta diversity were compared by HIV infection status for maternal stool and breast milk. No significant differences were observed in Shannon or Simpson indices for maternal stool (Cohen’s d ≈ −0.38; 95% CI: −3.88 to 0.07) (**Figure 2A**) or breast milk (Cohen’s d ≈ 0.16; 95% CI: −1.05 to 2.88) (**Figure 2B**) when stratified by HIV infection. However, HIV-infected mothers showed a non-significant trend toward lower alpha diversity in both maternal stool and breast milk compared with HIV-uninfected mothers. A similar trend was observed within the HIV-infected subgroup when alpha diversity was compared by cotrimoxazole use, with non-significantly lower diversity (Shannon, p = 0.32; Simpson, p = 0.23) among those taking cotrimoxazole (n = 12) compared with those not taking it (n = 13). No significant differences were observed for beta diversity (Bray–Curtis dissimilarity) in maternal stool (**Supplementary Figure 4A**) or breast milk (**Supplementary Figure 4B**) when compared by HIV status. Similarly, no differences in composition were observed within the HIV-infected subgroup when maternal stool (p = 0.386) or breast milk (p = 0.450) composition was compared by cotrimoxazole use.

In a sensitivity analysis excluding mothers on antibiotics, a non-significant trend toward lower alpha diversity persisted in the HIV-infected group. Beta diversity measures were similar when comparing these groups. Non-significant differences in alpha diversity were also observed in breast milk. However, a significant difference was observed for UniFrac distance (*p* = 0.013), but not for Bray–Curtis dissimilarity, when comparing HIV-uninfected and HIV-infected mothers not taking antibiotics. This likely reflects lineage-level differences rather than large shifts in relative abundances, with small sample size potentially amplifying metric discordance.

The infant gut microbiota was compared by HIV exposure status, and alpha diversity was similar between HEU infants and their HUU peers (Cohen’s d ≈ −0.21; 95% CI: −3.09 to 0.84) (**Figure 2C**). No significant differences were observed in infant stool composition (Bray–Curtis dissimilarity) between HEU and HUU infants (**Supplementary Figure 4C**). We also assessed potential effects of prophylactic antibiotics taken by HIV-infected women on infant gut microbiota. Given the higher prevalence of antibiotic use (46.2%) among HIV-infected women, this analysis was restricted to HEU infants. HEU infants born to women taking prophylactic antibiotics exhibited a significantly higher stool Simpson index (*p* = 0.022).

We further investigated the effects of infant morbidity on the infant gut microbiota. Overall, no significant effects of infant morbidity on stool alpha diversity were observed. Comparisons of infant stool beta diversity by morbidity status (**Supplementary Figure 5A**) and illness type (**Supplementary Figure 5B**) showed no significant differences (*p* > 0.05), including after Bonferroni correction for multiple testing. However, significant differences in infant stool beta diversity were observed by illness count among infants reporting illness (**Supplementary Figure 5C**; *p* = 0.019 and *p* = 0.02 before and after multiple testing correction). This suggests that overall illness burden may be more influential than specific conditions. The absence of effects for infant age, sex, weight, and length may reflect limited statistical power rather than evidence of no effect. Infant age effects were further examined by stratifying infants into ≤7 days and >7 days; no significant differences in Shannon or Simpson indices or beta diversity were observed between age groups. No differences in alpha or beta diversity were observed when stratified by infant sex.

### Taxa differences in mother stool, breast milk and infant stool compared by HIV status

**Figure 3:**
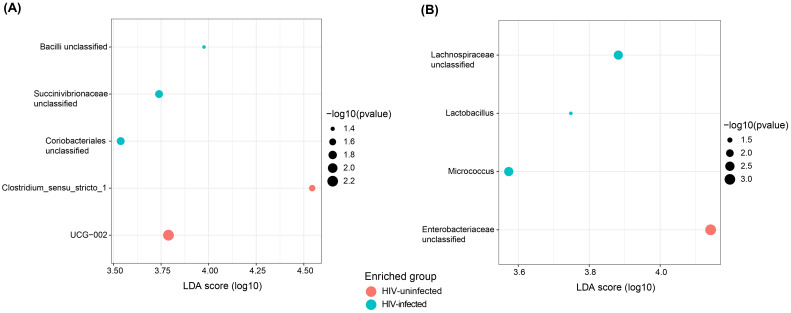
Taxa differences in mother stool and breast milk by HIV infection status. Comparisons of differentially abundant taxa in **(A)** mother stool and **(B)** breast milk by HIV infection status. LDA, linear discriminant analysis.

To identify abundant taxa across all sample types by HIV infection and exposure status, linear discriminant analysis effect size (LEfSe) was used with a linear discriminant analysis (LDA) cutoff of 3.5. Only comparisons significant at *p* < 0.05 were reported. Comparison of taxa in maternal stool by HIV status (**Figure 3A**) showed enrichment of *Clostridium_sensu_stricto_1 and UCG_002* from the *Ruminococcaceae* family in the HIV-uninfected group. Bacilli_*unclassified* and Coriobacteriales_*unclassified* were enriched in stool from HIV-infected mothers. Breast milk showed enrichment of *Enterobacteriaceae_unclassified* in HIV-uninfected mothers, whereas *Lactobacillus* and *Lachnospiraceae_unclassified* were enriched in HIV-infected mothers (**Figure 3B**). In infant stool, enrichment of *Clostridium_sensu_stricto_1* was noted in the HIV-exposed group. Further stratification of the already small sample size into HIV groups may reduce the ability to detect small differences in taxa, and interpretations should therefore be made with caution. The reported enrichments of *Lactobacillus* in breast milk of HIV-infected mothers and *Clostridium_sensu_stricto_1* in HIV-exposed uninfected (HEU) infants are intriguing, but their biological or clinical significance remains unclear. Previous studies have observed similar shifts in microbial composition in HIV contexts, yet consistently emphasize the need to clarify whether such changes are protective, compensatory, or detrimental to infant health (González et al., 2013; Endika et al., 2023; Tobin et al., 2024; Devarajalu et al., 2025).

MaAsLin2 analyses assessing the effects of HIV exposure, infant age, weight, length, delivery mode, and illness burden did not identify any taxa significantly associated with these variables (q > 0.05). Stratification of the analysis by HIV exposure status yielded the same result, with no detectable associations. These null findings likely reflect limited statistical power to detect small or moderate effect sizes rather than the absence of underlying biological relationships.

### Shared core taxa between breast milk and infant stool microbiota

**Figure 4:**
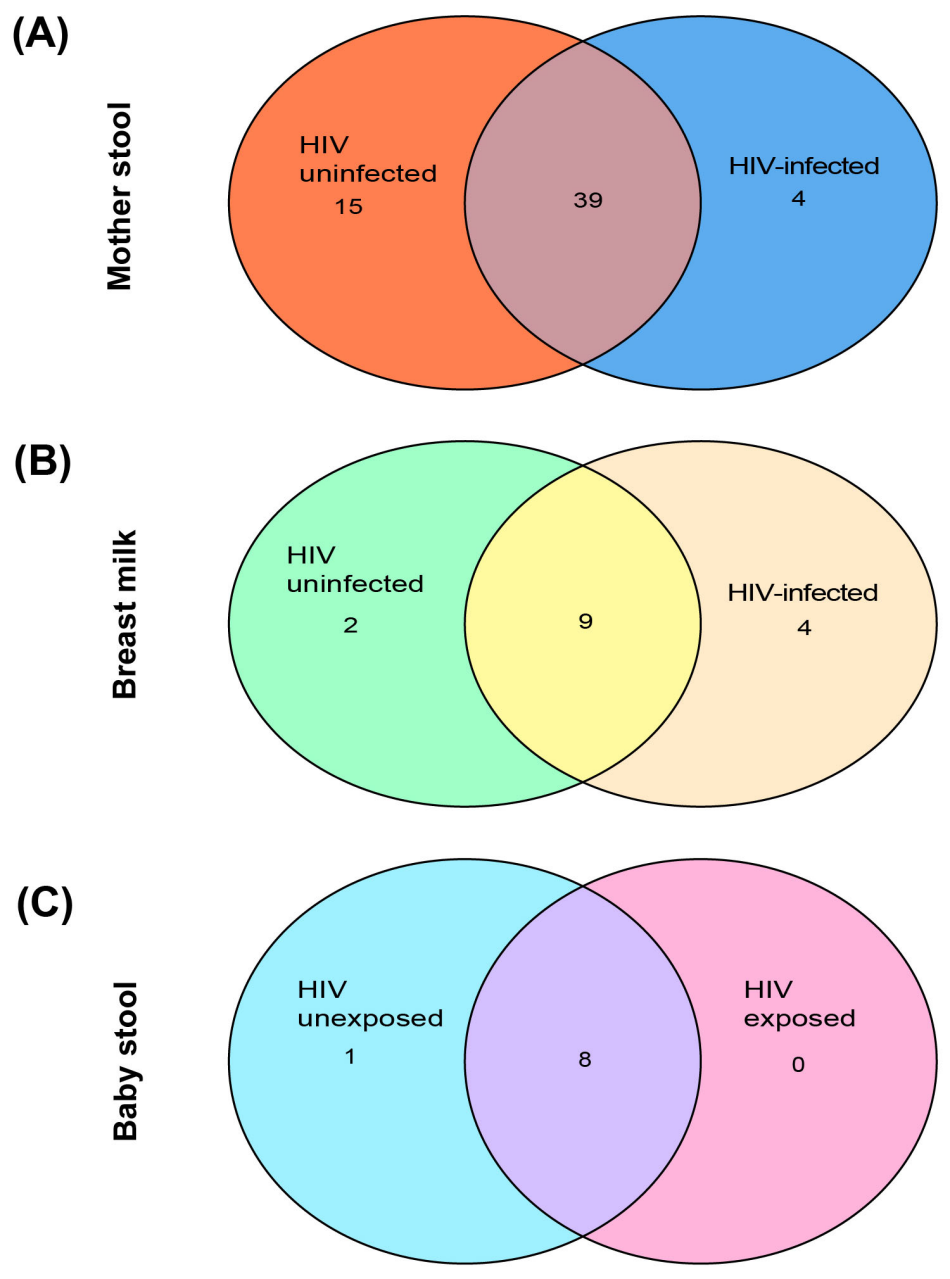
Core taxa comparisons by HIV infection and exposure status. Core taxa comparisons in **(A)** mother stool, **(B)** breast milk, and **(C)** infant stool by HIV infection and maternal HIV exposure status.

Core taxa were defined as taxa with a minimum relative abundance of ≥0.1% and present in at least 50% of samples. A total of nine core taxa were identified in infant stool, 15 core taxa in breast milk, and 58 core taxa in maternal stool. Venn diagrams were developed to determine the distribution of core taxa in maternal stool, breast milk, and infant stool by HIV infection and exposure status (**Figure 4**; **Supplementary Table 1**). Infant stool core taxa included *Streptococcus*, *Bifidobacterium*, and *Enterobacteriaceae*_*unclassified*, with some taxa shared between the HEU and HIV-unexposed uninfected (HUU) groups. *Coriobacteriaceae*_*unclassified* was noted in HIV-unexposed infants. In breast milk, *Streptococcus* and *Gemella* were among the shared core taxa by maternal HIV status. In maternal stool, *Prevotella, Solobacterium, Enterobacteriaceae_unclassified*, and Coriobacteriales_*unclassified* were found in the HIV-infected group, whereas *Eubacterium_coprostanoligenes_group*, *Ruminococcaceae_unclassified*, *UCG_002*, and *UCG_005* were noted in the HIV-uninfected group (**Supplementary Table 1**). Core taxa shared between breast milk and infant stool included *Streptococcus* and Bacillales_*unclassified*.

Of the 62 breast milk samples analyzed, 60 could be paired with corresponding infant stool samples. These pairs included breast milk samples from 23/60 (38.3%) HIV-infected women and 37/60 (61.7%) HIV-uninfected women. Core taxa shared between breast milk and infant stool were identified, and microbial source tracking analysis was used to estimate the proportion of infant gut taxa attributed to breast milk as a potential source. The analysis was conducted for paired breast milk and infant stool samples to estimate direct microbial contributions within mother–infant pairs. Overall, 31.5% of taxa observed in infant stool were estimated to have originated from breast milk, whereas 68.5% were attributed to other sources (*p* < 0.001). Further stratification by infant HIV exposure status showed that in HEU infants, 41% of the infant gut microbiota was estimated to originate from breast milk, while 59% was attributed to other sources (*p* = 0.259). In the HUU group, breast milk was estimated to contribute 25.6% of the infant gut microbiota, whereas 74.4% was acquired from other sources (*p* = 0.001).

### Core taxa correlated between breast milk and infant stool

**Figure 5:**
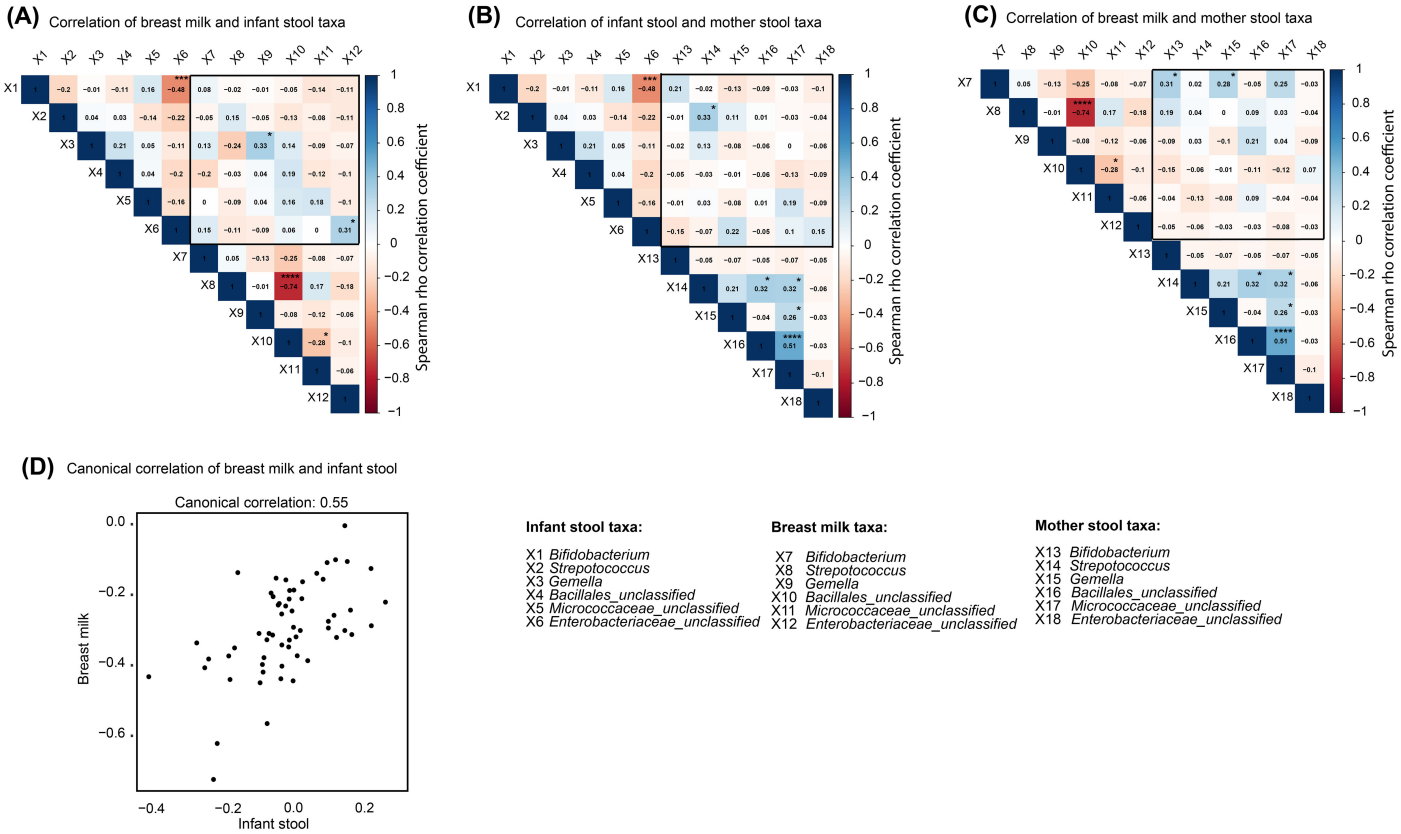
Correlation of mother stool, breast milk, and infant stool core taxa. Spearman’s rho correlation coefficients for core taxa comparisons in **(A)** breast milk versus infant stool, **(B)** infant stool versus mother stool, and **(C)** breast milk versus mother stool. **(D)** Canonical correlation between breast milk and infant stool, where each point represents canonical scores for a paired sample. Unadjusted p-value key: ****p<0.0001, ***p<0.001, **<0.01. *p<0.05.

A correlation matrix was generated for core taxa in breast milk against corresponding taxa in infant stool. Overall, positive correlations were observed for *Gemella* (*ρ* = 0.33, *p* = 0.010, adjusted *p* = 0.081) and *Enterobacteriaceae_unclassified* (*ρ* = 0.31, *p* = 0.017, adjusted *p* = 0.077) between breast milk and infant stool (**Figure 5A**). Negative correlations were also observed for *Bifidobacterium* versus *Enterobacteriaceae_unclassified* within infant stool (*ρ* = −0.48, *p* < 0.001, adjusted *p* = 0.001) and for *Streptococcus* versus Bacillales_*unclassified* within breast milk (*ρ* = −0.74, *p* < 0.001, adjusted *p* < 0.001). Correlations between maternal stool and infant stool core taxa showed a positive association for *Streptococcus* (*ρ* = 0.33, *p* = 0.011, adjusted *p* = 0.425). Additional positive correlations were observed among maternal stool core taxa, including a moderately strong correlation (*ρ* = 0.51, *p* < 0.001, adjusted *p* < 0.001) between Bacillales_*unclassified* and *Micrococcaceae_unclassified* (**Figure 5B**).

A similar analysis comparing maternal stool and breast milk identified a positive correlation for *Bifidobacterium* (*ρ* = 0.31, *p* = 0.012, adjusted *p =* 0.081). In addition, *Gemella* in maternal stool correlated positively (*ρ* = 0.28, *p* = 0.028, adjusted *p* = 0.264) with *Bifidobacterium* in breast milk (**Figure 5C**). These correlations do not demonstrate direct microbial transfer and may also reflect shared environmental sources. Relationships between core microbial taxa in breast milk and infant stool were further characterized using canonical correlation analysis (CCA) of relative abundances. This analysis indicated a moderate linkage between breast milk and infant stool core microbiomes between 7 and 14 days postpartum (**Figure 5D**). HIV infection/exposure, maternal age, infant age, and antibiotic use were overlaid onto the canonical scores; however, no clustering was observed across these variables, suggesting that none strongly structured the canonical relationships. R code for the CCA analysis (Supplementary File 1) was deposited in Figshare, with the link provided in the “Availability of data and materials” section.

## Discussion

The early-life gut microbiota is shaped by a combination of maternal, environmental, and infant-specific factors, and perturbations during this period can influence immune maturation and metabolic development. In this study, we evaluated how maternal HIV infection and postpartum antibiotic exposure relate to gut and breast milk microbial patterns during the first two weeks postpartum. As expected, maternal stool and breast milk exhibited higher microbial richness than infant stool, reflecting the mature and relatively stable maternal microbiome compared with the simpler, early colonizing communities in newborns. Early gut colonization is strongly influenced by maternal factors, including HIV infection, mode of delivery, antibiotic exposure, diet, and breastfeeding practices. In this study, maternal HIV status did not significantly affect microbial diversity or community composition in maternal stool or breast milk, nor did it strongly influence infant stool microbiota. Because HIV status in this cohort was closely linked to cotrimoxazole use, maternal age, socioeconomic stressors, and delivery mode, residual confounding from these factors may have influenced microbial patterns despite statistical adjustment. Similarly, prophylactic cotrimoxazole use among HIV-infected mothers did not produce significant differences in alpha or beta diversity in either maternal or infant samples. Breast milk remained a key contributor to the infant gut microbiota, with SourceTracker estimating that 31.5% of infant gut bacterial composition was derived from breast milk during the early postpartum period. Overall, these findings suggest that maternal HIV infection and infant HIV exposure do not strongly alter breast milk or early infant gut microbial diversity in this population, while confirming the central role of breast milk in seeding early-life microbiota.

### Maternal HIV infection status and maternal stool microbiota

The maternal stool microbiota was dominated by Bacillota, Bacteroidota, and Actinomycetota, with increased abundance of obligate anaerobes such as *Clostridium_sensu_stricto_1* and *Romboutsia* (Drell et al., 2017; Munjoma et al., 2023). In the same population during pregnancy, significant differences in stool alpha and beta diversity were observed between HIV-infected and HIV-uninfected mothers (Chandiwana et al., 2023), suggesting that pregnancy-associated immune and metabolic shifts may partly explain those earlier findings. In contrast, during the postpartum period analyzed here, no significant diversity differences by HIV status were detected, consistent with findings from Nigeria (Grant-Beurmann et al., 2022). Other studies have reported clear alterations in gut microbial richness and composition in HIV-infected adults, even under ART (Ling et al., 2016; Zhou et al., 2020; Munjoma et al., 2023), supporting an established link between HIV-associated immune dysfunction and microbial dysbiosis (Nowak et al., 2015; Ling et al., 2016; Jackson et al., 2022). In our dataset, multivariable regression models similarly did not identify significant associations between HIV status and maternal stool microbial composition. However, the absence of significant diversity differences should be interpreted cautiously, as the modest sample size limits statistical power and may mask subtle but biologically relevant HIV-associated microbial shifts. Notably, taxa-level differences observed in this study did not align with α- or β-diversity patterns and were not significant in multivariable MaAsLin2 models, further suggesting that these represent modest trends rather than broad community shifts.

Differential abundance analyses indicated enrichment of *Clostridium_sensu_stricto_1* and *UCG_002* in HIV-uninfected compared with HIV-infected mothers, consistent with earlier findings in the same cohort at 6 weeks and 6 months postpartum (Munjoma et al., 2023; Munjoma et al., 2024). In contrast, other studies have reported depletion of *Bacteroides, Bifidobacterium, Faecalibacterium*, and *Parabacteroides*, with increased *Prevotella* in HIV-infected individuals (Lozupone et al., 2013; Lu et al., 2018; Zhou et al., 2020), patterns not observed in this study. Instead, Bacilli, Coriobacteriales_*unclassified*, and *Succinivibrionaceae_unclassified* were modestly enriched in HIV-infected mothers, differing from previously reported HIV-associated microbial signatures. The increased abundance of *UCG*_*002* (class Clostridia) in HIV-uninfected mothers parallels findings in HIV-uninfected Japanese men who have sex with men, who showed increased *Clostridium*_*UCG-014* (Ishizaka et al., 2021). These taxa-level differences did not correspond to shifts in α- or β-diversity, and none remained significant in MaAsLin2 models, indicating that they likely represent isolated trends rather than broad community alterations. The metabolic role of *Clostridium_sensu_stricto_1*, including involvement in butyrate production and carbohydrate metabolism, may suggest functional enrichment in HIV-uninfected individuals. Differences across studies may reflect variation in HIV disease stage, ART regimens, environmental exposures, diet, socioeconomic factors, or methodological differences in sampling and sequencing. Overall, these taxa-level differences were modest in magnitude and did not correspond to significant associations in our diversity or MaAsLin2 models, suggesting that their biological relevance should be interpreted cautiously given the limited sample size.

In infant stool, *Enterobacteriaceae*_*unclassified*, *Streptococcus*, and *Bifidobacterium* were the most abundant taxa, consistent with findings in Nigerian infants at 6 months postpartum, where *Bifidobacterium* and *Streptococcus* dominated (Grant-Beurmann et al., 2022). The coexistence of *Bifidobacterium* and *Enterobacteriaceae* is characteristic of early microbial succession, in which bifidobacteria promote immune tolerance and metabolic regulation. Experimental evidence suggests that *Bifidobacterium* can competitively restrain *Enterobacteriaceae* through nutrient competition, pH reduction, and antimicrobial metabolite production (Sadeghpour Heravi and Hu, 2023; Samarra et al., 2024; Zou et al., 2025), indicating that these associations may reflect ecological competition rather than dysbiosis. Our findings differed from a Brazilian study at 6 weeks postpartum, where *Bacteroides* remained dominant regardless of HIV exposure status (Machiavelli et al., 2019), highlighting population-specific colonization trajectories.

Exposure to maternal HIV infection and postpartum antibiotics did not significantly alter infant gut microbiota diversity, consistent with findings from Brazil and South Africa at 4–6 weeks postpartum (Machiavelli et al., 2019; Jackson et al., 2022; Byrne et al., 2024). However, lower alpha diversity has been reported among Haitian and Zimbabwean HIV-exposed uninfected (HEU) infants (Bender et al., 2016; Flygel et al., 2020; Robertson et al., 2023), indicating that the impact of HIV exposure may vary across regions and contexts. In this study, HEU infants showed enrichment of *Clostridium_sensu_stricto_1* compared with HIV-unexposed uninfected (HUU) infants, a finding that differs from reports in South African HEU infants, who showed increased abundance of *Blautia, Klebsiella*, and *Ruminococcaceae* (Jackson et al., 2022; Johnson et al., 2025). Cameroonian HEU infants similarly exhibited increased prevalence of opportunistic pathogens, contributing to elevated morbidity (Nkenfou et al., 2019). In Brazilian HEU infants, enrichment of *Prevotella copri, Klebsiella*, and *Dorea* has been reported (Machiavelli et al., 2019), while South African HEU infants showed greater abundance of *Shigella flexneri*, *Shigella boydii*, and *Klebsiella pneumoniae* at 4 weeks postpartum (Byrne et al., 2024). Nigerian HEU infants have also demonstrated reduced *Bifidobacterium* levels and distinct shifts in alpha diversity up to 6 months postpartum (Grant-Beurmann et al., 2022). *Clostridium_sensu_stricto_1* is an early anaerobic colonizer involved in carbohydrate fermentation, but its enrichment in HEU infants remains difficult to contextualize biologically. Unlike the pathogen-enriched profiles reported in Cameroonian or South African HEU cohorts, the health implications of this pattern are uncertain and require confirmation in larger longitudinal studies. These inconsistencies across studies likely reflect differences in breastfeeding practices, environmental exposures, maternal diet, ART regimens, timing of sampling, and infant feeding patterns.

### Maternal prenatal and infant antibiotic use did not affect maternal or infant gut microbiota

Maternal prenatal and intrapartum antibiotic use has been associated with potential long-term effects on maternal and infant gut microbiota. In this study, more than 50% of HIV-infected mothers were receiving cotrimoxazole prophylaxis, yet diversity metrics did not differ significantly by antibiotic use. Interpretation remains cautious given the small sample size and potential loss of statistical power after subgrouping. Contrary to expectations, infant gut microbial diversity was not reduced by maternal antibiotic exposure; in fact, richness was higher among infants born to mothers receiving cotrimoxazole, possibly reflecting increased detection of rare taxa. In models adjusted for HIV exposure and antibiotic use, no significant associations were identified. These results differ from Canadian findings showing reduced microbial richness at 3 months postpartum (Azad et al., 2016; Chen et al., 2021) and from U.S. studies indicating reduced abundances of specific taxa following infant antibiotic use up to 2 years (Sugino et al., 2021) of age. Additionally, a Dutch study linked infant antibiotic use to lower *Bacteroides* and *Bifidobacterium* abundance (Penders et al., 2006). Our analysis suggests that antibiotic effects may depend on underlying maternal or infant factors not assessed in this study. Notably, infants experiencing more than one illness episode exhibited shifts in gut microbial composition, indicating that illness burden itself may contribute to variation in early microbiota.

### Maternal HIV infection and antibiotic use did not alter breast milk microbiota

Our study found maternal stool microbiota to be richer and more diverse than breast milk and infant stool microbiota, aligning with findings from Swiss, Mexican, Croatian, and Russian studies conducted during early postpartum periods (Jost et al., 2014; Williams et al., 2019; Corona-Cervantes et al., 2020; Banić et al., 2022). Limited data exist on the composition of the breast milk microbiota in low- and middle-income settings, particularly in Zimbabwe. Breast milk, rich in microbes, nutrients, and bioactive molecules, promotes a healthy infant gut by increasing commensal taxa such as *Bifidobacterium*, *Lactobacillus*, and *Bacteroides* (Le Doare et al., 2018; Williams et al., 2019; Carr et al., 2021; Mallick et al., 2021). In this study, the increased abundance of the phyla Bacillota, Actinomycetota, Bacteroidota, and Pseudomonadota in breast milk was consistent with findings from Mexican, European, American, and Chinese studies up to 6 months postpartum (Williams et al., 2017; Hermansson et al., 2019; Corona-Cervantes et al., 2020; Banić et al., 2022; Huang et al., 2022; Li et al., 2022). Alpha and beta diversity of breast milk microbiota showed no significant differences between HIV-infected and HIV-uninfected mothers, consistent with studies from South Africa, Zambia, and Haiti (Bender et al., 2016; Jackson et al., 2022; Tobin et al., 2024). These findings should be interpreted cautiously, as modest sample size, sequencing depth, and unmeasured maternal factors may have obscured smaller but biologically relevant differences.

In this study, the presence of *Streptococcus*, *Gemella*, and *Pseudomonas* in breast milk was consistent with findings from South African and Chinese studies conducted within the first month postpartum (Ge et al., 2021; Ge et al., 2021; Huang et al., 2022; Jackson et al., 2022; Li et al., 2022; Lyons et al., 2022; Wallenborn et al., 2022). Consistent with earlier reports, *Streptococcus* was among the most abundant taxa in breast milk, underscoring its role in maternal–infant microbial transfer and early gut colonization (Dombrowska-Pali et al., 2024). The dominance of *Streptococcus* in breast milk may be partly attributable to non-aseptic milk collection methods, potentially reflecting contributions from areolar skin or retrograde flow from the infant’s oral cavity (Le Doare et al., 2018; Kordy et al., 2020; Granger et al., 2021). HIV-infected mothers showed increased abundance of *Lachnospiraceae_unclassified* and *Micrococcus* compared with HIV-uninfected mothers, consistent with a South African study (Jackson et al., 2022). In contrast, studies from Haiti and Zambia reported increased abundance of *Acinetobacter*, *Rothia*, and *Gemella* in breast milk from HIV-infected mothers compared with HIV-uninfected mothers (Tobin et al., 2024). These discrepancies across cohorts likely reflect differences in sampling methods, sequencing workflows, maternal diet, ART regimens, and environmental exposures rather than contradictory biological effects.

The effect of maternal antibiotic use on breast milk microbiota was also examined and found not to significantly alter microbial diversity in the HIV-infected group. Because our assessment focused primarily on alpha and beta diversity, subtle antibiotic- or ART-associated shifts in specific taxa may not have been detected. In addition, the effects of combination antiretroviral therapy could not be evaluated, as all HIV-infected mothers were receiving the same triple-drug regimen (Tenolam-E). Our findings are consistent with a Kenyan study reporting non-significantly lower breast milk microbial diversity associated with antibiotic use and combination ART (Maqsood et al., 2022). Delivery mode and infant feeding practices are also known to influence breast milk and infant gut microbiota composition (Haddad et al., 2022; Ma et al., 2022); however, given the predominance of vaginal delivery and exclusive breastfeeding in this cohort, these effects were not discernible. Nonetheless, because cesarean delivery was associated with HIV exposure in this study, residual confounding may have masked potential HIV-related effects.

### Core taxa were correlated between breast milk and infant gut microbiota

Breast milk is naturally enriched in taxa such as *Streptococcus, Pseudomonas*, and *Staphylococcus*, whereas the infant gut in early life is typically dominated by *Bacteroides* and *Bifidobacterium* (Fehr et al., 2020; Lyons et al., 2020). In this study, we observed high abundances of *Streptococcus* and *Enterobacteriaceae*_*unclassified* in both breast milk and infant stool, potentially reflecting microbial transfer via retrograde flow or maternal skin contact. These pathways remain speculative in our dataset and are based on mechanisms demonstrated in previous experimental and observational studies rather than direct evidence from this analysis. Non-aseptic milk collection may also have contributed to overrepresentation of skin-associated taxa. Unlike Canadian and Finnish studies reporting strong correlations between *Streptococcus* and *Bifidobacterium* in mother–infant pairs (Grönlund et al., 2007; Murphy et al., 2017; Fehr et al., 2020), we observed weaker correlations, suggesting population-specific microbial transfer dynamics. These weaker associations may also reflect methodological factors, limited sample size, the early sampling window (7–14 days postpartum), and unmeasured maternal influences, and therefore may represent study limitations rather than true biological differences. Nonetheless, positive correlations for *Gemella* and *Enterobacteriaceae_unclassified* support shared maternal–infant microbial exchange, although these associations indicate microbial relatedness rather than definitive evidence of direct transmission.

Our analysis further indicated that a greater proportion of the gut microbiota in HIV-exposed uninfected (HEU) infants than in HIV-unexposed uninfected (HUU) infants was attributable to breast milk, consistent with findings from Brazilian and Haitian cohorts (Bender et al., 2016; Machiavelli et al., 2019). *SourceTracker* provides probabilistic estimates based on relative abundances and is sensitive to filtering thresholds, sequencing depth, and source definitions. In this study, samples with ≥2,000 reads were included without rarefaction. The estimated 31.5% contribution of breast milk to infant gut microbiota was lower than the 67.7% reported in Mexican infants and the 44.1% reported in Chinese infants (Corona-Cervantes et al., 2020; Ge et al., 2021). In an American cohort, 27.7% of the infant gut microbiota during the first month of life was estimated to originate from breast milk (Pannaraj et al., 2017). Differences across studies likely reflect variation in sampling time points, sequencing platforms, breast milk processing protocols, maternal diet, environmental exposures, and breastfeeding practices. The extent of microbial transfer may have functional consequences: acquisition of beneficial taxa such as *Bifidobacterium* can support immune maturation, whereas transfer of taxa such as *Streptococcus* may influence colonization dynamics and early infection risk. Larger longitudinal studies incorporating metagenomic and immunological profiling are needed to clarify the mechanisms and health implications of maternal–infant microbial transmission. Finally, these correlations and *SourceTracker* estimates should be interpreted cautiously given the modest sample size and limited adjustment for potential confounders, including maternal antibiotic use, delivery mode, and infant feeding practices.

## Strengths and limitations of the study

To our knowledge, this is one of the first studies to characterize the breast milk microbiota of Zimbabwean HIV-infected and uninfected lactating women, filling a critical gap in sub-Saharan Africa, where findings from high-income countries may not be generalizable. The paired sampling of breast milk and infant stool enabled a more reliable evaluation of maternal–infant microbial relationships, and the study holds clear clinical relevance given the importance of early microbial exposure for immune maturation, infection susceptibility, and growth trajectories.

Several limitations should be considered when interpreting the findings. The use of high-speed centrifugation for breast milk DNA extraction may underrepresent fat-associated bacteria that are potentially transmitted during breastfeeding (Stinson et al., 2021; Young et al., 2022), leading to an underestimation of maternal–infant microbial transfer. We did not assess maternal factors such as diet, human milk oligosaccharides (HMOs), secretor status, environmental exposures, or nutritional status, each of which can influence breast milk composition and shape the infant gut microbiota. Among HIV-infected women, all participants were receiving a uniform Tenolam-E regimen, preventing evaluation of variation in cART effects. Furthermore, the close link between HIV status and cotrimoxazole prophylaxis represents an unavoidable confounder, limiting our ability to disentangle their independent contributions. The narrow sampling window (7–14 days postpartum) provides an early snapshot but limits generalizability across infancy, as both breast milk and infant gut microbiota evolve substantially over the first 2–5 years of life. Larger longitudinal studies incorporating detailed maternal phenotyping and functional microbiome analyses are needed to fully understand the mechanisms mediating breast milk–infant microbial interactions in this context.

## Conclusion

Early postpartum maternal health and breastfeeding practices remain central determinants of infant gut microbiota assembly. In this study, we characterized the breast milk and infant gut microbiota of HIV-infected and uninfected lactating women and found no statistically significant differences in microbial richness or overall community composition by maternal HIV status or antibiotic prophylaxis. Given the modest sample size, potential unmeasured confounders, and reliance on diversity metrics, these findings should be interpreted cautiously, as small or moderate effects may have gone undetected.

We observed a higher estimated contribution of breast milk taxa to the infant gut among HIV-exposed infants, suggesting that maternal HIV infection could influence patterns of vertical microbial transfer even in the absence of major shifts in alpha or beta diversity. Because these estimates were derived from *SourceTracker*, which is sensitive to data structure, sequencing depth, and preprocessing decisions, this observation should be viewed as preliminary and hypothesis-generating rather than definitive.

Nevertheless, correlations between specific breast milk and infant gut taxa reinforce the broader concept that breast milk is a key contributor to early-life microbial colonization. If confirmed in larger cohorts, differences in the magnitude or composition of microbial transfer could have implications for infant immune maturation, susceptibility to infections, and metabolic development—processes known to be shaped by early microbial exposure.

Overall, our findings suggest that maternal HIV status may not strongly alter breast milk or infant gut microbial diversity at 7–14 days postpartum but may subtly modulate patterns of vertical microbial transmission. Further longitudinal studies with larger sample sizes and deeper multi-omics profiling are needed to clarify the biological significance of these early microbial differences for infant health.

## Data Availability

The datasets presented in this study can be found in online repositories. The names of the repository/repositories and accession number(s) can be found below:https://figshare.com/s/50e17c3e6c549ea99335, FigShare.

## Supplementary material

Supplementary Table 1 Core taxa in **(A)** mother stool, **(B)** breast milk, and **(C)** infant stool stratified by HIV infection and exposure status **Abbreviations:** HIV: Human Immunodeficiency Virus.

Supplementary Figure 1 Rarefaction curve for pooled samples. Rarefaction curves for pooled mother stool, breast milk, and infant stool showing **(A)** Shannon index and **(B)** Simpson index. Curves are color-coded by sample type

Supplementary Figure 2 Overall alpha and beta diversity in mother stool, breast milk, and infant stool. **(A)** Comparison of Shannon and Simpson indices in mother stool, breast milk, and infant stool. **(B)** Beta diversity (Bray–Curtis dissimilarity) in mother stool, breast milk, and infant stool. Key: **** = p<0.0001, *** = p<0.001, ** = p<0.01. * = p<0.05

Supplementary Figure 3 Taxonomy profile for identified genera by HIV infection and exposure status. Pie charts comparing relative abundances of the top 10 most abundant identified genera in **(A)** mother stool, **(B)** breast milk, and **(C)** infant stool stratified by HIV infection and maternal HIV exposure status.

Supplementary Figure 4 Similar beta diversity by HIV infection and exposure status. Comparison of beta diversity indices (Bray–Curtis dissimilarity) in **(A)** mother stool, **(B)** breast milk, and **(C)** infant stool by HIV infection and maternal HIV exposure status. PCoA, principal coordinates analysis

Supplementary Figure 5 Infant stool beta diversity comparison by illness type and count: **(A)** PCoA plot comparing infant stool beta diversity by illness on or before the visit date. **(B)** PCoA plot comparing infant stool beta diversity by illness type, and **(C)** by illness count.

## Glossary

AIDS: Acquired Immunodeficiency Syndrome
BCG: Bacille Calmette-Guerin
BP: Blood Pressure
cART: combination Antiretroviral Therapy
CCA: Canonical Correlation Analysis
DNA: Deoxyribonucleic Acid
EDTA: Ethylenediaminetetraacetic Acid
EID: Early Infant Diagnosis
FDR: False Discovery Rate
HEI: HIV-exposed Infected
HEU: HIV-exposed uninfected
HIV: Human Immunodeficiency Virus
HMOs: Human Milk Oligosaccharides
HUU: HIV-unexposed uninfected
IQR: Interquartile Range
LDA: Linear Discriminant Analysis
MUAC: Mid Upper Arm Circumference
PCoA: Principal Coordinate Analysis
PCR: Polymerase Chain Reaction
PERMANOVA: Permutational Multivariate Analysis of Variance
QIIME2: Quantitative Insights into Microbial Ecology 2
REDCap: Research Electronic Data Capture
rRNA: ribosomal Ribonucleic Acid
SD: Standard Deviation
USD: United States Dollar
UZBCS: University of Zimbabwe Birth Cohort Study
WHO: World Health Organisation.

## References

[B1] Arbizu P. M. (2024). pmartinezarbizu/pairwiseAdonis. Available online at: https://github.com/pmartinezarbizu/pairwiseAdonis (Accessed January 24, 2025).

[B2] (2023). Update of recommendations on first- and second-line antiretroviral regimens. Available online at: https://www.who.int/publications-detail-redirect/WHO-CDS-HIV-19.15 (Accessed February 3, 2025).

[B3] (2024). R: the R project for statistical computing. Available online at: https://www.r-project.org/ (Accessed January 8, 2025).

[B4] Azad M. Konya T. Persaud R. Guttman D. Chari R. Field C. . (2016). Impact of maternal intrapartum antibiotics, method of birth and breastfeeding on gut microbiota during the first year of life: a prospective cohort study. BJOG Int. J. Obstet Gynaecol. 123, 983–993. doi: 10.1111/1471-0528.13601, PMID: 26412384

[B5] Banić M. Butorac K. Čuljak N. Leboš Pavunc A. Novak J. Bellich B. . (2022). The human milk microbiota produces potential therapeutic biomolecules and shapes the intestinal microbiota of infants. Int. J. Mol. Sci. 23, 14382. doi: 10.3390/ijms232214382, PMID: 36430861 PMC9699365

[B6] Bender J. M. Li F. Martelly S. Byrt E. Rouzier V. Leo M. . (2016). Maternal HIV infection influences the microbiome of HIV-uninfected infants. Sci. Transl. Med. 8, 349ra100–349ra100. doi: 10.1126/scitranslmed.aaf5103, PMID: 27464748 PMC5301310

[B7] Bolyen E. Rideout J. R. Dillon M. R. Bokulich N. A. Abnet C. C. Al-Ghalith G. A. . (2019). Reproducible, interactive, scalable and extensible microbiome data science using QIIME 2. Nat. Biotechnol. 37, 852–857. doi: 10.1038/s41587-019-0209-9, PMID: 31341288 PMC7015180

[B8] Boudry G. Charton E. Le Huerou-Luron I. Ferret-Bernard S. Le Gall S. Even S. . (2021). The relationship between breast milk components and the infant gut microbiota. Front. Nutr. 8, 629740. doi: 10.3389/fnut.2021.629740, PMID: 33829032 PMC8019723

[B9] Byrne A. Diener C. Brown B. P. Maust B. S. Feng C. Alinde B. L. . (2024). Neonates exposed to HIV but uninfected exhibit an altered gut microbiota and inflammation associated with impaired breast milk antibody function. Microbiome. 12, 261. doi: 10.1186/s40168-024-01973-z, PMID: 39707483 PMC11662858

[B10] Callahan B. J. Sankaran K. Fukuyama J. A. McMurdie P. J. Holmes S. P. (2016). Bioconductor Workflow for Microbiome Data Analysis: from raw reads to community analyses. F1000Research. 5, 1492. doi: 10.12688/f1000research.8986.2, PMID: 27508062 PMC4955027

[B11] Caporaso J. G. Kuczynski J. Stombaugh J. Bittinger K. Bushman F. D. Costello E. K. . (2010). QIIME allows analysis of high-throughput community sequencing data. Nat. Methods 7, 335–336. doi: 10.1038/nmeth.f.303, PMID: 20383131 PMC3156573

[B12] Carr L. E. Virmani M. D. Rosa F. Munblit D. Matazel K. S. Elolimy A. A. . (2021). Role of human milk bioactives on infants’ Gut and immune health. Front. Immunol. 12, 604080. doi: 10.3389/fimmu.2021.604080/full, PMID: 33643310 PMC7909314

[B13] Chandiwana P. Munjoma P. T. Mazhandu A. J. Li J. Baertschi I. Wyss J. . (2023). Antenatal gut microbiome profiles and effect on pregnancy outcome in HIV infected and HIV uninfected women in a resource limited setting. BMC Microbiol. 23, 4. doi: 10.1186/s12866-022-02747-z, PMID: 36604616 PMC9817306

[B14] Cheema A. S. Trevenen M. L. Turlach B. A. Furst A. J. Roman A. S. Bode L. . (2022). Exclusively breastfed infant microbiota develops over time and is associated with human milk oligosaccharide intakes. Int. J. Mol. Sci. 23, 2804. doi: 10.3390/ijms23052804, PMID: 35269946 PMC8910998

[B15] Chen Y. Y. Zhao X. Moeder W. Tun H. M. Simons E. Mandhane P. J. . (2021). Impact of maternal intrapartum antibiotics, and caesarean section with and without labour on bifidobacterium and other infant gut microbiota. Microorganisms. 9, 1847. doi: 10.3390/microorganisms9091847, PMID: 34576741 PMC8467529

[B16] Chong H. Y. Tan L. T. H. Law J. W. F. Hong K. W. Ratnasingam V. Ab Mutalib N. S. . (2022). Exploring the potential of human milk and formula milk on infants’ Gut and health. Nutrients. 14, 3554. doi: 10.3390/nu14173554, PMID: 36079814 PMC9460722

[B17] Corona-Cervantes K. García-González I. Villalobos-Flores L. E. Hernández-Quiroz F. Piña-Escobedo A. Hoyo-Vadillo C. . (2020). Human milk microbiota associated with early colonization of the neonatal gut in Mexican newborns. PeerJ. 8, e9205. doi: 10.7717/peerj.9205, PMID: 32509465 PMC7247532

[B18] Cortes-Macías E. Selma-Royo M. García-Mantrana I. Calatayud M. González S. Martínez-Costa C. . (2021). Maternal diet shapes the breast milk microbiota composition and diversity: impact of mode of delivery and antibiotic exposure. J. Nutr. 151, 330–340. doi: 10.1093/jn/nxaa310, PMID: 33188413 PMC7850106

[B19] Custer G. F. Gans M. van Diepen L. T. A. Dini-Andreote F. Buerkle C. A. (2023). Comparative analysis of core microbiome assignments: implications for ecological synthesis. mSystems. 8, e01066–e01022. doi: 10.1128/msystems.01066-22, PMID: 36744955 PMC9948721

[B20] Devarajalu P. Kumar J. Dutta S. Attri S. V. Kabeerdoss J. (2025). Gut microbiota alteration in healthy preterm infants: an observational study from tertiary care center in India. Microorganisms. 13, 577. doi: 10.3390/microorganisms13030577, PMID: 40142471 PMC11944540

[B21] Diakhate M. M. Unger J. A. Langat A. Singa B. Kinuthia J. Itindi J. . (2024). Factors associated with exclusive breastfeeding by maternal HIV status: a population-based survey in Kenya. Int. Breastfeed J. 19, 44. doi: 10.1186/s13006-024-00651-y, PMID: 38926772 PMC11210159

[B22] Ding J. Ouyang R. Zheng S. Wang Y. Huang Y. Ma X. . (2022). Effect of breastmilk microbiota and sialylated oligosaccharides on the colonization of infant gut microbial community and fecal metabolome. Metabolites. 12, 1136. doi: 10.3390/metabo12111136, PMID: 36422276 PMC9698434

[B23] Dinleyici M. Barbieur,.J. D. Cagri E. Vandenplas Y. (2023). Functional effects of human milk oligosaccharides (HMOs). Gut Microbes 15, 2186115. doi: 10.1080/19490976.2023.2186115, PMID: 36929926 PMC10026937

[B24] Dombrowska-Pali A. Wiktorczyk-Kapischke N. Chrustek A. Olszewska-Słonina D. Gospodarek-Komkowska E. Socha M. W. (2024). Human milk microbiome—A review of scientific reports. Nutrients. 16, 1420. doi: 10.3390/nu16101420, PMID: 38794658 PMC11124344

[B25] Drell T. Štšepetova J. Simm J. Rull K. Aleksejeva A. Antson A. . (2017). The influence of different maternal microbial communities on the development of infant gut and oral microbiota. Sci. Rep. 7, 1–9. doi: 10.1038/s41598-017-09278-y, PMID: 28855595 PMC5577157

[B26] Duri K. Gumbo F. Z. Munjoma P. T. Chandiwana P. Mhandire K. Ziruma A. . (2020). The University of Zimbabwe College of Health Sciences (UZ-CHS) BIRTH COHORT study: rationale, design and methods. BMC Infect. Dis. 20, 725. doi: 10.1186/s12879-020-05432-6, PMID: 33008316 PMC7532096

[B27] Endika M. F. Barnett D. J. M. Klostermann C. E. Schols H. A. Arts I. C. W. Penders J. . (2023). Microbiota-dependent influence of prebiotics on the resilience of infant gut microbiota to amoxicillin/clavulanate perturbation in an *in vitro* colon model. Front. Microbiol. 14. doi: 10.3389/fmicb.2023.1131953/full, PMID: 37275167 PMC10232780

[B28] Fehr K. Moossavi S. Sbihi H. Boutin R. C. T. Bode L. Robertson B. . (2020). Breastmilk feeding practices are associated with the co-occurrence of bacteria in mothers’ Milk and the infant gut: the CHILD cohort study. Cell Host Microbe 28, 285–297.e4. doi: 10.1016/j.chom.2020.06.009, PMID: 32652062

[B29] Flygel T. T. Sovershaeva E. Claassen-Weitz S. Hjerde E. Mwaikono K. S. Odland JØ . (2020). Composition of gut microbiota of children and adolescents with perinatal human immunodeficiency virus infection taking antiretroviral therapy in Zimbabwe. J. Infect. Dis. 221, 483–492. doi: 10.1093/infdis/jiz473, PMID: 31549151 PMC7457326

[B30] Froń A. Orczyk-Pawiłowicz M. (2024). Breastfeeding beyond six months: evidence of child health benefits. Nutrients. 16, 3891. doi: 10.3390/nu16223891, PMID: 39599677 PMC11597163

[B31] Ge Y. Zhu W. Chen L. Li D. Li Q. Jie H. (2021). The maternal milk microbiome in mammals of different types and its potential role in the neonatal gut microbiota composition. Animals. 11, 3349. doi: 10.3390/ani11123349, PMID: 34944125 PMC8698027

[B32] González R. Mandomando I. Fumadó V. Sacoor C. Macete E. Alonso P. L. . (2013). Breast milk and gut microbiota in african mothers and infants from an area of high HIV prevalence. PloS One 8, e80299. doi: 10.1371/journal.pone.0080299, PMID: 24303004 PMC3841168

[B33] Granger C. L. Embleton N. D. Palmer J. M. Lamb C. A. Berrington J. E. Stewart C. J. (2021). Maternal breastmilk, infant gut microbiome and the impact on preterm infant health. Acta Paediatr. 110, 450–457. doi: 10.1111/apa.15534, PMID: 33245565

[B34] Grant-Beurmann S. Jumare J. Ndembi N. Matthew O. Shutt A. Omoigberale A. . (2022). Dynamics of the infant gut microbiota in the first 18 months of life: the impact of maternal HIV infection and breastfeeding. Microbiome. 10, 61. doi: 10.1186/s40168-022-01230-1, PMID: 35414043 PMC9004197

[B35] Gregory K. E. Samuel B. S. Houghteling P. Shan G. Ausubel F. M. Sadreyev R. I. . (2016). Influence of maternal breast milk ingestion on acquisition of the intestinal microbiome in preterm infants. Microbiome. 4, 68. doi: 10.1186/s40168-016-0214-x, PMID: 28034306 PMC5200970

[B36] Grönlund M. M. Gueimonde M. Laitinen K. Kociubinski G. Grönroos T. Salminen S. . (2007). Maternal breast-milk and intestinal bifidobacteria guide the compositional development of the Bifidobacterium microbiota in infants at risk of allergic disease. Clin. Exp. Allergy 37, 1764–1772. doi: 10.1111/j.1365-2222.2007.02849.x, PMID: 17941914

[B37] Haddad E. N. Ferro L. E. Russell K. E. B. Sugino K. Y. Kerver J. M. Comstock S. S. (2022). Fecal bacterial communities differ by lactation status in postpartum women and their infants. J. Hum. Lact. 38, 270–280. doi: 10.1177/08903344211060343, PMID: 34903081

[B38] Harris P. A. Taylor R. Minor B. L. Elliott V. Fernandez M. O’Neal L. . (2019). The REDCap consortium: Building an international community of software platform partners. J. BioMed. Inform. 95, 103208. doi: 10.1016/j.jbi.2019.103208, PMID: 31078660 PMC7254481

[B39] Hermansson H. Kumar H. Collado M. C. Salminen S. Isolauri E. Rautava S. (2019). Breast milk microbiota is shaped by mode of delivery and intrapartum antibiotic exposure. Front. Nutr. 6. doi: 10.3389/fnut.2019.00004, PMID: 30778389 PMC6369203

[B40] Huang T. Zeng Z. Liang X. Tang X. Luo H. Wang D. . (2022). Effect of breast milk with or without bacteria on infant gut microbiota. BMC Pregnancy Childbirth. 22, 1–11. doi: 10.1186/s12884-022-04930-6, PMID: 35883060 PMC9317457

[B41] Ishizaka A. Koga M. Mizutani T. Parbie P. K. Prawisuda D. Yusa N. . (2021). Unique gut microbiome in HIV patients on antiretroviral therapy (ART) suggests association with chronic inflammation. Microbiol. Spectr. 9, e0070821. doi: 10.1128/Spectrum.00708-21, PMID: 34378948 PMC8552706

[B42] Jackson C. L. Frank D. N. Robertson C. E. Ir D. Kofonow J. M. Montlha M. P. . (2022). Evolution of the gut microbiome in HIV-exposed uninfected and unexposed infants during the first year of life. mBio. 13, e01229–e01222. doi: 10.1128/mbio.01229-22, PMID: 36073815 PMC9600264

[B43] Johnson M. J. Lazarus S. K. Bennett A. E. Tovar-Salazar A. Robertson C. E. Kofonow J. M. . (2025). Gut microbiota and other factors associated with increased T cell regulation in HIV-exposed uninfected infants. Front. Immunol. 16. doi: 10.3389/fimmu.2025.1533003/full, PMID: 40098966 PMC11911520

[B44] Jost T. Lacroix C. Braegger C. Chassard C. (2015). Impact of human milk bacteria and oligosaccharides on neonatal gut microbiota establishment and gut health. Nutr. Rev. 73, 426–437. doi: 10.1093/nutrit/nuu016, PMID: 26081453

[B45] Jost T. Lacroix C. Braegger C. P. Rochat F. Chassard C. (2014). Vertical mother–neonate transfer of maternal gut bacteria via breastfeeding. Environ. Microbiol. 16, 2891–2904. doi: 10.1111/1462-2920.12238, PMID: 24033881

[B46] Knights D. Kuczynski J. Charlson E. S. Zaneveld J. Mozer M. C. Collman R. G. . (2011). Bayesian community-wide culture-independent microbial source tracking. Nat. Methods 8, 761–763. doi: 10.1038/nmeth.1650, PMID: 21765408 PMC3791591

[B47] Kordy K. Gaufin T. Mwangi M. Li F. Cerini C. Lee D. J. . (2020). Contributions to human breast milk microbiome and enteromammary transfer of Bifidobacterium breve. PloS One 15, e0219633. doi: 10.1371/journal.pone.0219633, PMID: 31990909 PMC6986747

[B48] Laursen M. F. Pekmez C. T. Larsson M. W. Lind M. V. Yonemitsu C. Larnkjær A. . (2021). Maternal milk microbiota and oligosaccharides contribute to the infant gut microbiota assembly. ISME Commun. 1, 21. doi: 10.1038/s43705-021-00021-3, PMID: 36737495 PMC9723702

[B49] Le Doare K. Holder B. Bassett A. Pannaraj P. S. (2018). Mother’s milk: A purposeful contribution to the development of the infant microbiota and immunity. Front. Immunol. 9. doi: 10.3389/fimmu.2018.00361/full, PMID: 29599768 PMC5863526

[B50] Li Y. Ren L. Wang Y. Li J. Zhou Q. Peng C. . (2022). The effect of breast milk microbiota on the composition of infant gut microbiota: A cohort study. Nutrients. 14, 5397. doi: 10.3390/nu14245397, PMID: 36558556 PMC9781472

[B51] Ling Z. Jin C. Xie T. Cheng Y. Li L. Wu N. (2016). Alterations in the fecal microbiota of patients with HIV-1 infection: an observational study in A chinese population. Sci. Rep. 6, 1–12. doi: 10.1038/srep30673, PMID: 27477587 PMC4967929

[B52] Liu F. He S. Yan J. Yan S. Chen J. Lu Z. . (2022). Longitudinal changes of human milk oligosaccharides, breastmilk microbiome and infant gut microbiome are associated with maternal characteristics. Int. J. Food Sci. Technol. 57, 2793–2807. doi: 10.1111/ijfs.15324

[B53] Lozupone C. A. Li M. Campbell T. B. Flores S. C. Linderman D. Gebert M. J. . (2013). Alterations in the gut microbiota associated with HIV-1 infection. Cell Host Microbe 14, 329–339. doi: 10.1016/j.chom.2013.08.006, PMID: 24034618 PMC3864811

[B54] Lu W. Feng Y. Jing F. Han Y. Lyu N. Liu F. . (2018). Association Between Gut Microbiota and CD4 Recovery in HIV-1 Infected Patients. Front. Microbiol. 9. doi: 10.3389/fmicb.2018.01451/full, PMID: 30034377 PMC6043814

[B55] Lyons K. E. CAO’ S. Grimaud G. CA R. Dempsey E. AL K. . (2022). The human milk microbiome aligns with lactation stage and not birth mode. Sci. Rep. 12, 5598. doi: 10.1038/s41598-022-09009-y, PMID: 35379843 PMC8979980

[B56] Lyons K. E. Ryan C. A. Dempsey E. M. Ross R. P. Stanton C. (2020). Breast milk, a source of beneficial microbes and associated benefits for infant health. Nutrients. 12, 1039. doi: 10.3390/nu12041039, PMID: 32283875 PMC7231147

[B57] Ma J. Li Z. Zhang W. Zhang C. Zhang Y. Mei H. . (2022). Comparison of the gut microbiota in healthy infants with different delivery modes and feeding types: A cohort study. Front. Microbiol. 13, 868227. doi: 10.3389/fmicb.2022.868227/full, PMID: 35722310 PMC9204251

[B58] Machiavelli A. Duarte R. T. D. Pires MM de S. Zárate-Bladés C. R. Pinto A. R. (2019). The impact of in *utero* HIV exposure on gut microbiota, inflammation, and microbial translocation. Gut Microbes 10, 599–614. doi: 10.1080/19490976.2018.1560768, PMID: 30657007 PMC6748604

[B59] Mallick H. Rahnavard A. McIver L. J. Ma S. Zhang Y. Nguyen L. H. . (2021). Multivariable association discovery in population-scale meta-omics studies. PloS Comput. Biol. 17, e1009442. doi: 10.1371/journal.pcbi.1009442, PMID: 34784344 PMC8714082

[B60] Maqsood R. Skidmore P. T. Holland L. A. Au J. L. Khan A. K. Wu L. I. . (2022). Dynamic changes in breast milk microbiome in the early postpartum period of Kenyan women living with HIV are influenced by antibiotics but not antiretrovirals. Microbiol. Spectr. 10, e02080–e02021. doi: 10.1128/spectrum.02080-21, PMID: 35384692 PMC9045247

[B61] Martínez-Martínez M. Martínez-Martínez M. Soria-Guerra R. Gamiño-Gutiérrez S. Senés-Guerrero C. Santacruz A. . (2024). Influence of feeding practices in the composition and functionality of infant gut microbiota and its relationship with health. PloS One 19, e0294494. doi: 10.1371/journal.pone.0294494, PMID: 38170702 PMC10763948

[B62] McMurdie P. J. Holmes S. (2013). phyloseq: an R package for reproducible interactive analysis and graphics of microbiome census data. PloS One 8, e61217. doi: 10.1371/journal.pone.0061217, PMID: 23630581 PMC3632530

[B63] Milani C. Duranti S. Bottacini F. Casey E. Turroni F. Mahony J. . (2017). The first microbial colonizers of the human gut: composition, activities, and health implications of the infant gut microbiota. Microbiol. Mol. Biol. Rev. 81, e00036–e00017. doi: 10.1128/MMBR.00036-17, PMID: 29118049 PMC5706746

[B64] Munjoma P. T. Chandiwana P. Wyss J. Mazhandu A. J. Jordi S. B. U. Gutsire R. . (2023). Immune activation and inflammation in lactating women on combination antiretroviral therapy: role of gut dysfunction and gut microbiota imbalance. Front. Immunol. 14. doi: 10.3389/fimmu.2023.1280262, PMID: 38045684 PMC10693333

[B65] Munjoma P. T. Mazhandu A. J. Wyss J. Jordi S. B. U. Leolin K. Yilmaz B. . (2024). Characterization of the gut microbiota and systemic inflammation in HIV-exposed uninfected infants from a resource-limited setting at 6 weeks of age. Microb. Health Dis. 6, e1156. doi: 10.26355/mhd_202411_1156

[B66] Murphy K. Curley D. O’Callaghan T. F. O’Shea C. A. Dempsey E. M. O’Toole P. W. . (2017). The composition of human milk and infant faecal microbiota over the first three months of life: A pilot study. Sci. Rep. 7, 40597. doi: 10.1038/srep40597, PMID: 28094284 PMC5240090

[B67] Nkenfou C. N. Abange W. B. Gonsu H. K. Kamgaing N. Lyonga E. M. Anoubissi J de D. . (2019). Evaluation of the effect of HIV virus on the digestive flora of infected versus non infected infants. Pan Afr Med. J. 34, 24. doi: 10.11604/pamj.2019.34.24.15039, PMID: 31762893 PMC6859050

[B68] Nowak P. Troseid M. Avershina E. Barqasho B. Neogi U. Holm K. . (2015). Gut microbiota diversity predicts immune status in HIV-1 infection. AIDS Lond Engl. 29, 2409–2418. doi: 10.1097/QAD.0000000000000869, PMID: 26355675

[B69] Pace R. M. Williams J. E. Robertson B. Lackey K. A. Meehan C. L. Price W. J. . (2021). Variation in human milk composition is related to differences in milk and infant fecal microbial communities. Microorganisms. 9, 1153. doi: 10.3390/microorganisms9061153, PMID: 34072117 PMC8230061

[B70] Padilha M. Danneskiold-Samsøe N. B. Brejnrod A. Hoffmann C. Cabral V. P. Iaucci J de M. . (2019). The human milk microbiota is modulated by maternal diet. Microorganisms. 7, 502. doi: 10.3390/microorganisms7110502, PMID: 31671720 PMC6920866

[B71] Pannaraj P. S. Li F. Cerini C. Bender J. M. Yang S. Rollie A. . (2017). Association between breast milk bacterial communities and establishment and development of the infant gut microbiome. JAMA Pediatr. 171, 647–654. doi: 10.1001/jamapediatrics.2017.0378, PMID: 28492938 PMC5710346

[B72] Penders J. Thijs C. Vink C. Stelma F. F. Snijders B. Kummeling I. . (2006). Factors influencing the composition of the intestinal microbiota in early infancy. Pediatrics. 118, 511–521. doi: 10.1542/peds.2005-2824, PMID: 16882802

[B73] Pheeha S. M. Tamuzi J. L. Chale-Matsau B. Manda S. Nyasulu P. S. (2023). A scoping review evaluating the current state of gut microbiota research in africa. Microorganisms. 11, 2118. doi: 10.3390/microorganisms11082118, PMID: 37630678 PMC10458939

[B74] Quast C. Pruesse E. Yilmaz P. Gerken J. Schweer T. Yarza P. . (2013). The SILVA ribosomal RNA gene database project: improved data processing and web-based tools. Nucleic Acids Res. 41, D590–D596. doi: 10.1093/nar/gks1219, PMID: 23193283 PMC3531112

[B75] Robertson R. C. Edens T. J. Carr L. Mutasa K. Gough E. K. Evans C. . (2023). The gut microbiome and early-life growth in a population with high prevalence of stunting. Nat. Commun. 14, 654. doi: 10.1038/s41467-023-36135-6, PMID: 36788215 PMC9929340

[B76] Sadeghpour Heravi F. Hu H. (2023). Bifidobacterium: host–microbiome interaction and mechanism of action in preventing common gut-microbiota-associated complications in preterm infants: A narrative review. Nutrients. 15, 709. doi: 10.3390/nu15030709, PMID: 36771414 PMC9919561

[B77] Samarra A. Cabrera-Rubio R. Martínez-Costa C. Collado M. C. (2024). The role of Bifidobacterium genus in modulating the neonate microbiota: implications for antibiotic resistance acquisition in early life. Gut Microbes 16, 2357176. doi: 10.1080/19490976.2024.2357176, PMID: 38798019 PMC11135851

[B78] Sánchez C. Fente C. Regal P. Lamas A. Lorenzo M. P. (2021). Human milk oligosaccharides (HMOs) and infant microbiota: A scoping review. Foods. 10, 1429. doi: 10.3390/foods10061429, PMID: 34203072 PMC8234547

[B79] Sharon I. Quijada N. M. Pasolli E. Fabbrini M. Vitali F. Agamennone V. . (2022). The core human microbiome: does it exist and how can we find it? A critical review of the concept. Nutrients. 14, 2872. doi: 10.3390/nu14142872, PMID: 35889831 PMC9323970

[B80] Stinson L. F. Ma J. Rea A. Dymock M. Geddes D. T. (2021). Centrifugation does not remove bacteria from the fat fraction of human milk. Sci. Rep. 11, 1–8. doi: 10.1038/s41598-020-79793-y, PMID: 33436707 PMC7804008

[B81] Sugino K. Y. Ma T. Paneth N. Comstock S. S. (2021). Effect of environmental exposures on the gut microbiota from early infancy to two years of age. Microorganisms. 9, 2140. doi: 10.3390/microorganisms9102140, PMID: 34683461 PMC8537618

[B82] Sun W. Tao L. Qian C. Pei X. P. Si D. Na T. Y. (2025). Human milk oligosaccharides: bridging the gap in intestinal microbiota between mothers and infants. Front. Cell Infect. Microbiol. 14. doi: 10.3389/fcimb.2024.1386421/full, PMID: 39835278 PMC11743518

[B83] Tobin N. H. Li F. Brummel S. Flynn P. M. Dababhai S. Moodley D. . (2024). Maternal HIV infection and the milk microbiome. Microbiome. 12, 1–15. doi: 10.1186/s40168-024-01843-8, PMID: 39342403 PMC11439335

[B84] Wallenborn J. T. Gunier R. B. Pappas D. J. Chevrier J. Eskenazi B. (2022). Breastmilk, stool, and meconium: bacterial communities in South Africa. Microb. Ecol. 83, 246–251. doi: 10.1007/s00248-021-01758-z, PMID: 33885917 PMC8531170

[B85] Wang K. Xia X. Sun L. Wang H. Li Q. Yang Z. . (2023). Microbial diversity and correlation between breast milk and the infant gut. Foods. 12, 1740. doi: 10.3390/foods12091740, PMID: 37174279 PMC10178105

[B86] Whiteley A. S. Jenkins S. Waite I. Kresoje N. Payne H. Mullan B. . (2012). Microbial 16S rRNA Ion Tag and community metagenome sequencing using the Ion Torrent (PGM) Platform. J. Microbiol. Methods 91, 80–88. doi: 10.1016/j.mimet.2012.07.008, PMID: 22849830

[B87] Williams J. E. Carrothers J. M. Lackey K. A. Beatty N. F. Brooker S. L. Peterson H. K. . (2019). Strong multivariate relations exist among milk, oral, and fecal microbiomes in mother-infant dyads during the first six months postpartum. J. Nutr. 149, 902–914. doi: 10.1093/jn/nxy299, PMID: 31063198 PMC6543206

[B88] Williams J. E. Carrothers J. M. Lackey K. A. Beatty N. F. York M. A. Brooker S. L. . (2017). Human milk microbial community structure is relatively sta ble and related to variations in macronutrient and micronutrient intakes in healthy lactating women. J. Nutr. 147, 1739–1748. doi: 10.3945/jn.117.248864, PMID: 28724659 PMC5572491

[B89] Yilmaz B. Fuhrer T. Morgenthaler D. Krupka N. Wang D. Spari D. . (2022). Plasticity of the adult human small intestinal stoma microbiota. Cell Host Microbe 30, 1773–1787.e6. doi: 10.1016/j.chom.2022.10.002, PMID: 36318918

[B90] Yilmaz B. Juillerat P. Øyås O. Ramon C. Bravo F. D. Franc Y. . (2019). Microbial network disturbances in relapsing refractory Crohn’s disease. Nat. Med. 25, 323–336. doi: 10.1038/s41591-018-0308-z, PMID: 30664783

[B91] Yilmaz B. Spalinger M. R. Biedermann L. Franc Y. Fournier N. Rossel J. B. . (2018). The presence of genetic risk variants within PTPN2 and PTPN22 is associated with intestinal microbiota alterations in Swiss IBD cohort patients. PloS One 13, e0199664. doi: 10.1371/journal.pone.0199664, PMID: 29965986 PMC6028086

[B92] Young G. R. Yew W. C. Nelson A. Bridge S. H. Berrington J. E. Embleton N. D. . (2022). Optimisation and application of a novel method to identify bacteriophages in maternal milk and infant stool identifies host-phage communities within preterm infant gut. Front. Pediatr. 10, 856520. doi: 10.3389/fped.2022.856520/full, PMID: 35558373 PMC9087270

[B93] Zhou D. T. Mudhluli T. E. Hall L. J. Manasa J. Munyati S. (2023). A scoping review of gut microbiome and bifidobacterium research in Zimbabwe: implications for future studies. Pediatr. Health Med. Ther. 14, 483–496. doi: 10.2147/PHMT.S414766, PMID: 38145055 PMC10743709

[B94] Zhou J. Zhang Y. Cui P. Luo L. Chen H. Liang B. . (2020). Gut microbiome changes associated with HIV infection and sexual orientation. Front. Cell Infect. Microbiol. 10. doi: 10.3389/fcimb.2020.00434/full, PMID: 33102244 PMC7546801

[B95] Zou X. Zhou X. Wang C. Zheng Y. Li Y. Suo H. (2025). The composition, influencing factors, and physiological functions of bifidobacteria in the infant gut: a review. Food Funct. 16, 7512–7530. doi: 10.1039/D5FO02271A, PMID: 40928706

