# Supplementary material for: The influence of breast milk microbiota from HIV-infected women on infant gut microbiota colonization within the first two weeks of life
Source: Front Microbiomes. 2026 Jan 28;5:1611702. doi: 10.3389/frmbi.2026.1611702 (PMC12993679; doi:10.3389/frmbi.2026.1611702)
Supplement: Supplementary Table 1 — Core taxa in (A) mother stool, (B) breast milk, and (C) infant stool stratified by HIV infection and exposure status Abbreviations: HIV: Human Immunodeficiency Virus. [file Table1.docx]

**Supplementary Table 1:** Core taxa in (A) mother stool, (B) breast milk and (C) infant stool stratified by HIV infection and exposure status

| **(A) Mother stool** | | |
| --- | --- | --- |
| **Core taxa**  **HIV-infected**  **(*n* = 4)** | **Shared core taxa between HIV-infected and HIV-uninfected**  **(*n* = 39)** | **Core taxa**  **HIV-uninfected**  **(*n* = 15)** |
| Prevotella | 2 x [Eubacterium]_hallii_group | [Eubacterium]_coprostanoligenes_  group |
| Solobacterium_metagenome | Agathobacter | Clostridium_sensu_stricto_1 |
| Enterobacteriaceae_unclassified | Anaerostipes | Holdemanella |
| Coriobacteriales_unclassified | 2 x Blautia | Turicibacter |
|  | Christensenellaceae_R-7_group | 2 x UCG-002 |
|  | Collinsella | UCG-005 |
|  | Dorea | Butyricicoccaceae_unclassified |
|  | 2 x Faecalibacterium | Erysipelotrichaceae_unclassified |
|  | Fusicatenibacter | Ruminococcaceae_unclassified |
|  | 2 x Marvinbryantia | 5 x Lachnospiraceae_unclassified |
|  | 2 x Romboutsia |  |
|  | Clostridium_sensu_stricto_1 |  |
|  | Subdoligranulum |  |
|  | Anaerovoracaceae_unclassified |  |
|  | Coriobacteriaceae_unclassified |  |
|  | Erysipelatoclostridiaceae_unclassified |  |
|  | 2 x Oscillospiraceae_unclassified |  |
|  | Peptostreptococcaceae_unclassified |  |
|  | 2 x Ruminococcaceae_unclassified |  |
|  | 12 x Lachnospiraceae_unclassified |  |
|  | Oscillospirales_unclassified |  |
| **(B) Mother milk** | | |
| **Core Taxa**  **HIV-infected**  **(*n* = 4)** | **Shared Core Taxa**  **(HIV-infected and HIV-uninfected) (*n* = 9)** | **Core Taxa**  **HIV-uninfected**  **(*n* = 2)** |
| Corynebacterium | Gemella | Gemella (different species) |
| Lactobacillus | 5 x Streptococcus | Streptococcus (different species) |
| Enterobacteriaceae_unclassified | Micrococcaceae_unclassified |  |
| Bacilli_unclassified | 2 x Bacillales_unclassified |  |
| **(C) Baby stool** | | |
| **Core Taxa**  **HIV-exposed**  **(*n* = 0)** | **Shared Core Taxa**  **(HIV-exposed and HIV-unexposed) (*n* = 8)** | **Core Taxa**  **HIV-unexposed**  **(*n* = 1)** |
|  | Bifidobacterium | Coriobacteriaceae_unclassified |
|  | 2 x Streptococcus |  |
|  | Enterobacterales_unclassified |  |
|  | 2 x Enterobacteriaceae_unclassified |  |
|  | 2 x Bacillales_unclassified |  |

**Abbreviations:** HIV: Human Immunodeficiency Virus**.**
