# Supplementary figures and images for: The influence of breast milk microbiota from HIV-infected women on infant gut microbiota colonization within the first two weeks of life
Source: Front Microbiomes. 2026 Jan 28;5:1611702. doi: 10.3389/frmbi.2026.1611702 (PMC12993679; doi:10.3389/frmbi.2026.1611702)

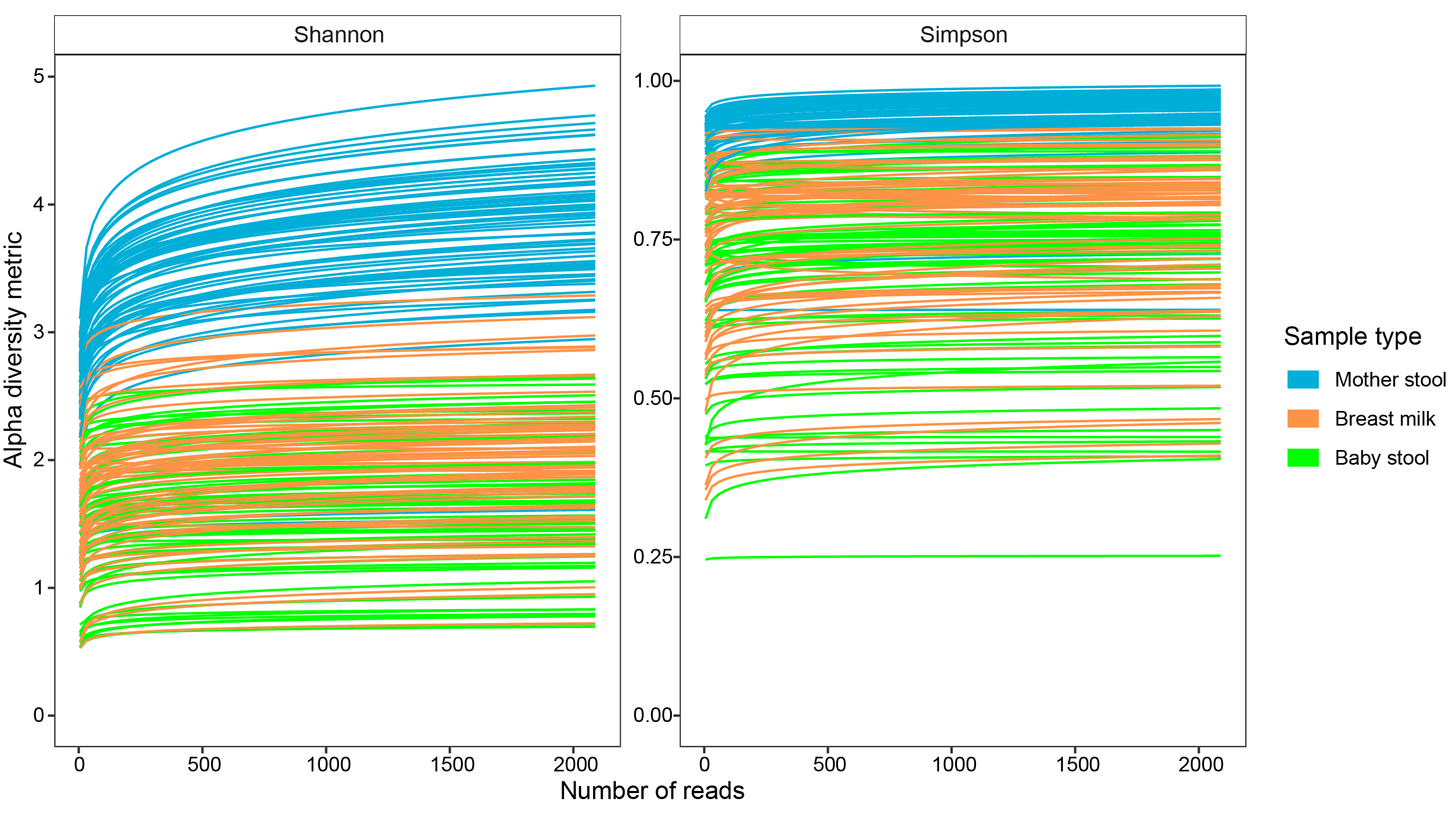

Supplement: Supplementary Figure 1 — Rarefaction curve for pooled samples. Rarefaction curves for pooled mother stool, breast milk, and infant stool showing (A) Shannon index and (B) Simpson index. Curves are color-coded by sample type [file Image1.jpeg]

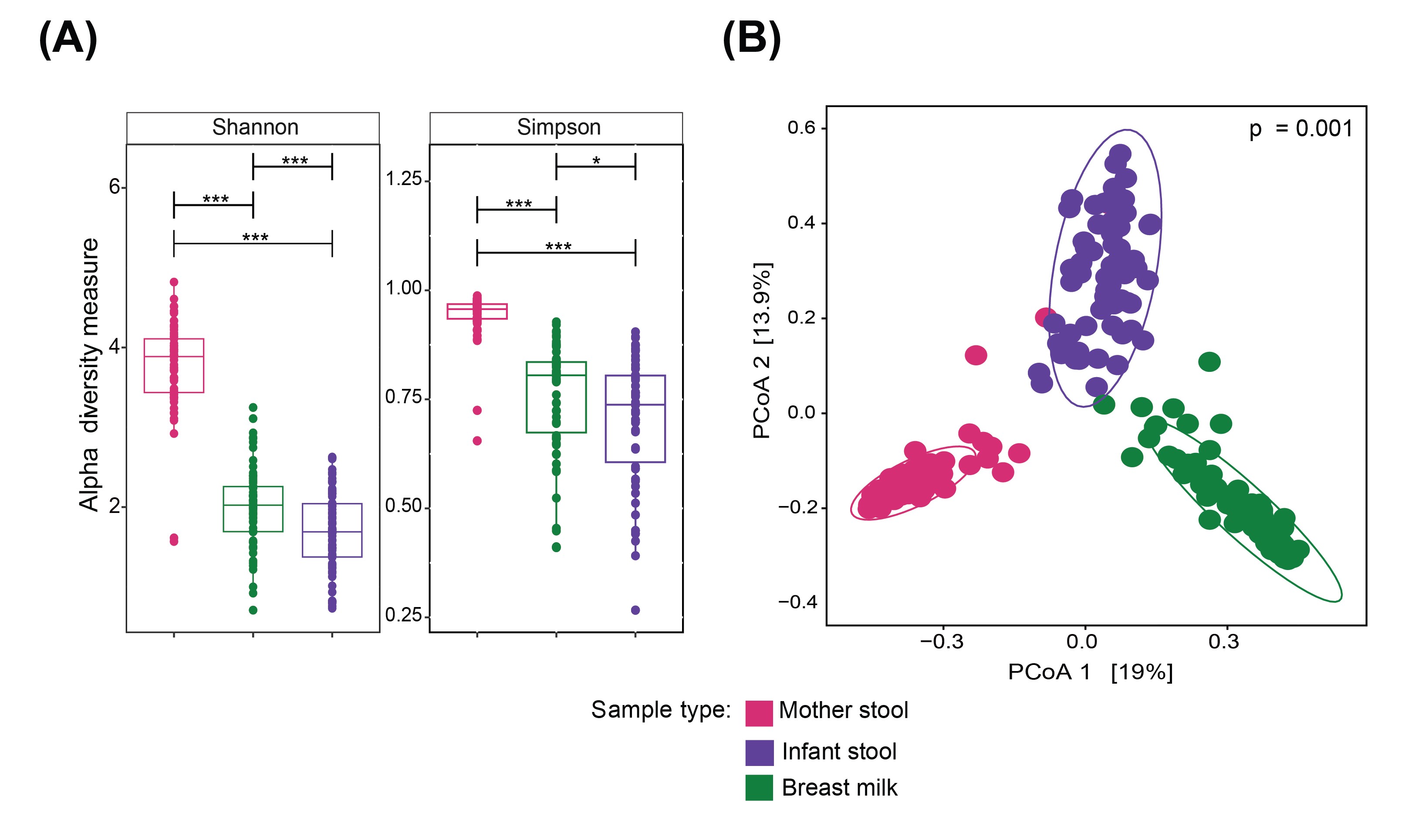

Supplement: Supplementary Figure 2 — Overall alpha and beta diversity in mother stool, breast milk, and infant stool. (A) Comparison of Shannon and Simpson indices in mother stool, breast milk, and infant stool. (B) Beta diversity (Bray–Curtis dissimilarity) in mother stool, breast milk, and infant stool. Key: **** = p<0.0001, *** = p<0.001, ** = p<0.01. * = p<0.05 [file Image2.jpeg]

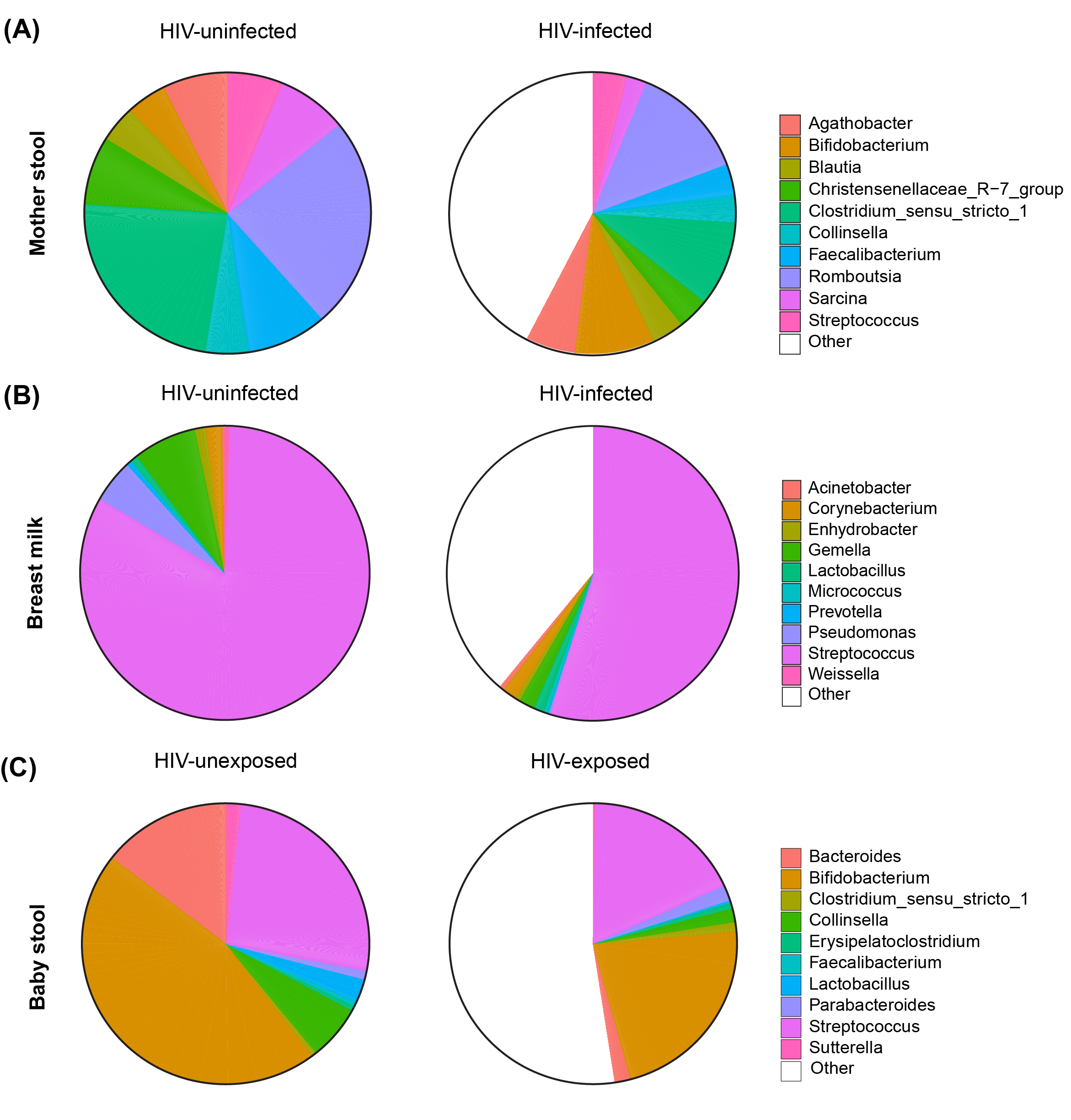

Supplement: Supplementary Figure 3 — Taxonomy profile for identified genera by HIV infection and exposure status. Pie charts comparing relative abundances of the top 10 most abundant identified genera in (A) mother stool, (B) breast milk, and (C) infant stool stratified by HIV infection and maternal HIV exposure status. [file Image3.jpeg]

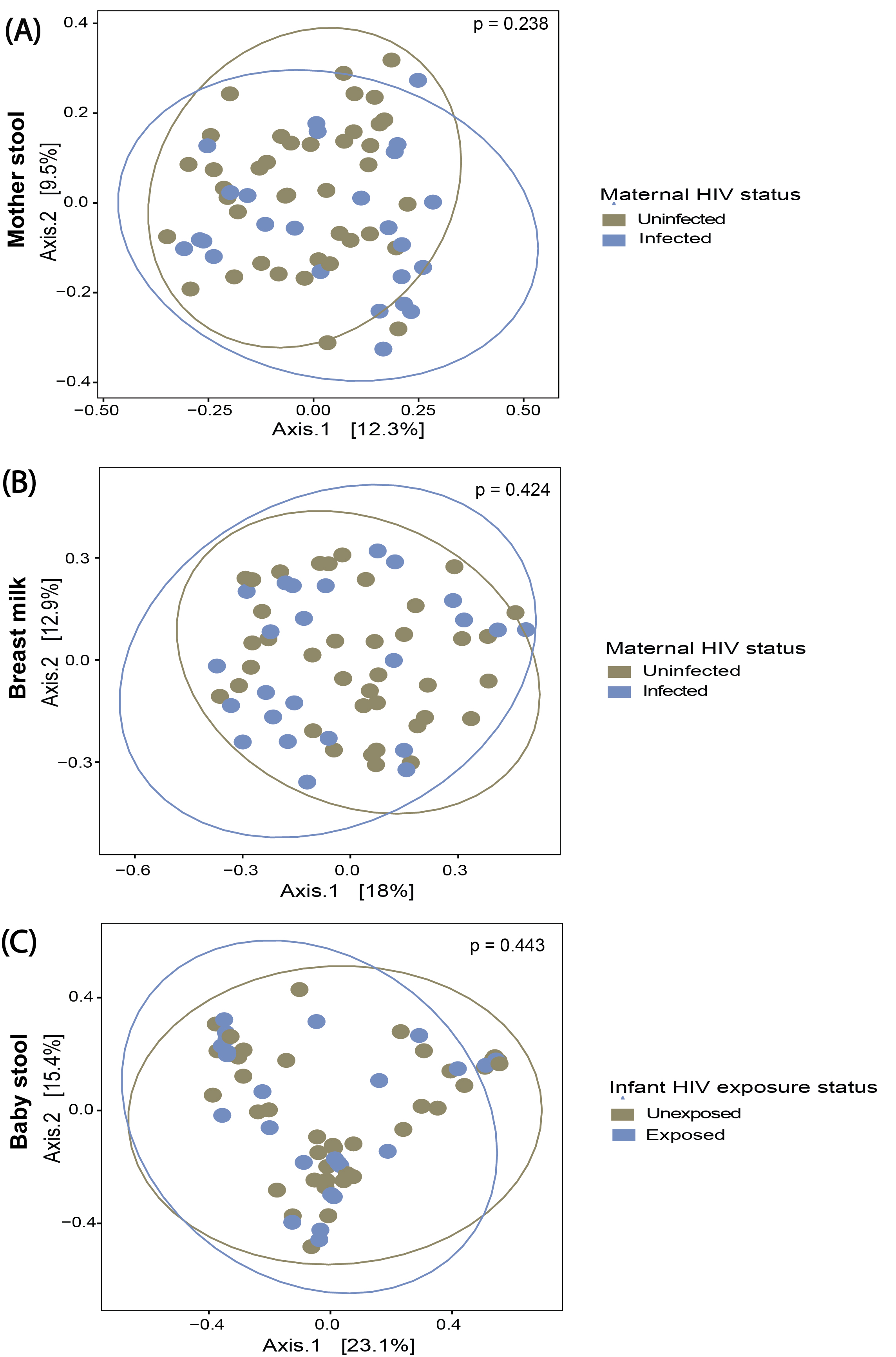

Supplement: Supplementary Figure 4 — Similar beta diversity by HIV infection and exposure status. Comparison of beta diversity indices (Bray–Curtis dissimilarity) in (A) mother stool, (B) breast milk, and (C) infant stool by HIV infection and maternal HIV exposure status. PCoA, principal coordinates analysis [file Image4.jpeg]

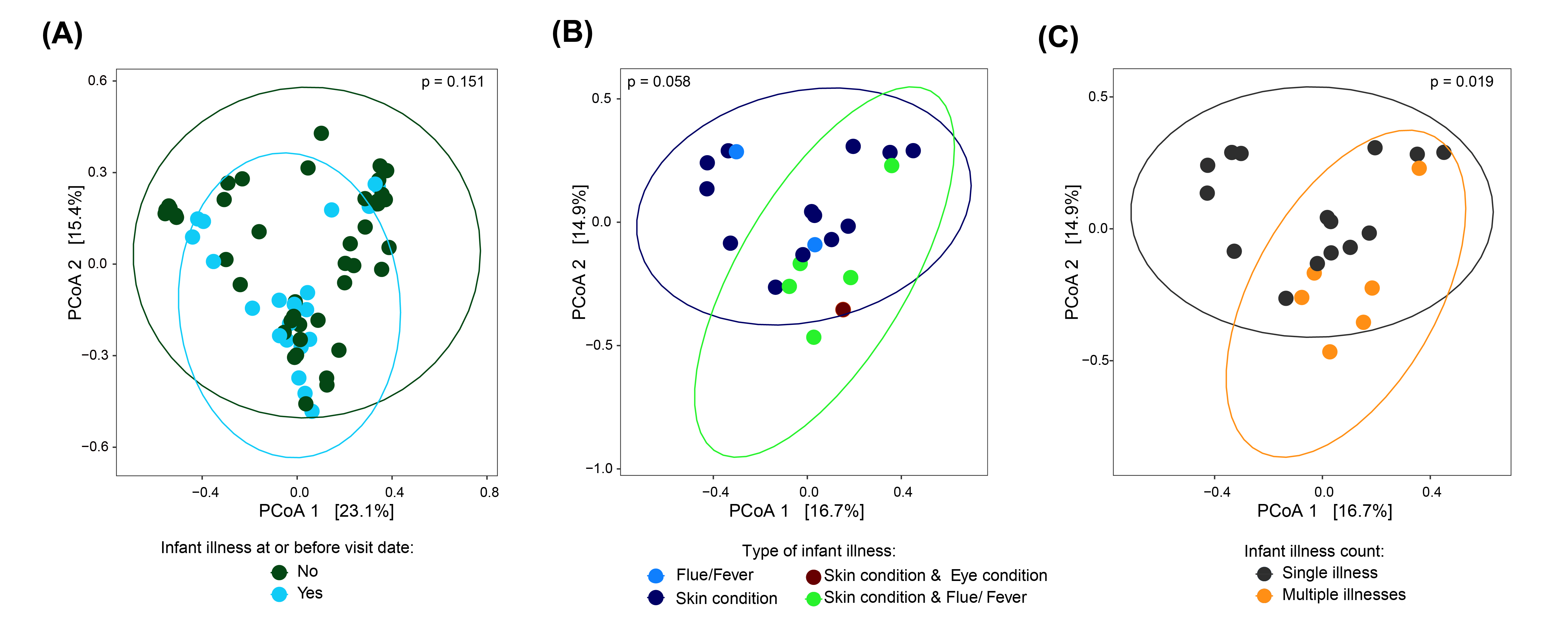

Supplement: Supplementary Figure 5 — Infant stool beta diversity comparison by illness type and count: (A) PCoA plot comparing infant stool beta diversity by illness on or before the visit date. (B) PCoA plot comparing infant stool beta diversity by illness type, and (C) by illness count. [file Image5.jpeg]
